# A comprehensive genome-wide profiling comparison between HBV and HCV infected hepatocellular carcinoma

**DOI:** 10.1186/s12920-019-0580-x

**Published:** 2019-10-28

**Authors:** Suofeng Sun, Yuan Li, Shuangyin Han, Hongtao Jia, Xiuling Li, Xiaofang Li

**Affiliations:** 1 Department of Gastroenterology, Henan Provincial People’s Hospital, People’s Hospital of Zhengzhou University, Zhengzhou, 450003 Henan China; 2 Department of Traditional Chinese Medicine, The Third Affiliated Hospital Affiliated of Henan University of Traditional Chinese Medicine, Zhengzhou, China; 3 Tianjia Genomes Tech CO, LTD., No. 6 Longquan Road, Anhui Chaohu economic develop zone, Hefei, 238014 People’s Republic of China

**Keywords:** Hepatocellular carcinoma, Hepatitis B virus, Hepatitis C virus, DNA methylation, Gene expression

## Abstract

**Background:**

Hepatocellular carcinoma (HCC) is one of the most common cancers worldwide, especially in East Asia. Even with the progress in therapy, 5-year survival rates remain unsatisfied. Chronic infection with the hepatitis B virus (HBV) or hepatitis C virus (HCV) has been epidemiologically associated with HCC and is the major etiology in the East Asian population. The detailed mechanism, especially the changes of DNA methylation and gene expression between the two types of virus-related HCC, and their contributions to the HCC development, metastasis, and recurrence remain largely unknown.

**Methods:**

In this integrated analysis, we characterized genome-scale profiles of HBV and HCV infected HCC by comparing their gene expression pattern, methylation profiles, and copy number variations from the publicly accessible data of The Cancer Genome Atlas Program (TCGA).

**Results:**

The HLA-A, STAT1, and OAS2 genes were highly enriched and up-regulated discovered in the HCV-infected HCC. Hypomethylation but not copy number variations might be the major factor for the up-regulation of these immune-related genes in HCV-infected HCC.

**Conclusions:**

The results indicated the different epigenetic changes of HBV/HCV related hepatocarcinogenesis. The top up-regulated genes in HCV group were significantly clustered in the immune-related and defense response pathways. These findings will help us to understand the pathogenesis of HBV/HCV associated hepatocellular carcinoma.

## Background

Hepatocellular carcinoma (HCC) is the most common type of primary liver cancer in adults and is the most common cause of death in people with cirrhosis [1]. Worldwide, this cancer is the third leading cause of cancer-related deaths, leading to about 1 million deaths annually [2]. 5-year survival rates of HCC remain unfavorable. For people at an early stage, the 5-year survival rate is about 31%, and if the tumor has spread, the 5-year survival rate can be as low as between 3 and 11% [3–6]. The disease arises in the hepatocytes, the cells that make up most of the liver. HCC is a heterogeneous disease, and in most cases, the etiologies are long-term damage and cirrhosis and are attributable to four major risk factors: infection with hepatitis B virus (HBV), infection with hepatitis C virus (HCV), chronic alcohol consumption, and exposure to aflatoxin B1 [7]. HCC is one of the most frequently occurring malignancies in Asian countries. The highest incidence occurs in Southeast Asia and is associated primarily with chronic HBV (China), or HCV (Japan) infection [8, 9].

The East Asian neonatal vaccination program has resulted in a tremendous decrease (70–85%) of the incidence of HBV-related HCC [10]. However, the total incidence increased recently and is expected to continue to escalate because of the global prevalence of nonalcoholic steatohepatitis (NASH) and HCV. It has been widely believed that the continuing epidemic of HCV largely accounts for the observed increase in HCC incidence [8, 11]. Despite the advances in medications recently, the survival rate did not improve much in the past two decades. Therefore, a better understanding of the underlying biological mechanisms involved in HCC pathogenesis and progression is critical for the development of novel diagnostic biomarkers and therapeutic strategies.

HBV and HCV are two of four major risk factors for HCC and are similar in both viral pathogens. However, the detailed differences between the two viruses regarding the pathogenesis of HCC remains unclear. HBV is a double-stranded, circular DNA molecule and transmitted via contaminated blood transfusions, intravenous injections, and sexual contact [12]. HCV is a small, single-stranded RNA virus that encodes a large polyprotein of about 3000 amino acid residues from a single open reading frame, which exhibits high genetic variability [13]. It will be beneficial for the mechanism studies to elucidate the genetic and epigenetic changes caused by the two viruses.

DNA methylation is a major event of epigenetic modifications and has been extensively investigated in recent cancer research [14]. A global DNA hypomethylation of oncogenes has been described as an almost universal finding in varieties of cancers [15, 16] and concurrent gene-specific hypermethylation has been observed at specific tumor-suppressor gene sites [16, 17]. DNA methylation is the major epigenetic feature of loci with main functions in gene transcriptional regulation as well as the preservation of genome stability. Wide varieties of malignancies are characterized by aberrancies in DNA methylation [18, 19]. Gene expression profiling and aberrant DNA methylation in HCC have been observed in previous studies. HBV CpG methylation has been reported to be significantly correlated with the hepatocarcinogenesis. While in HCV-infected HCC, the DNA methylation has also been suggested to play an important role by silencing tumor suppressors and might be used as a prognostic marker [20, 21].

With the progress of new techniques, the genome-level analysis provides a unique opportunity to study the mechanism of HBV and HCV pathogenesis, particularly for the HCC. The recent genome-wide DNA methylation profiling studies have revealed substantial DNA methylation changes in HCC [22–25]. However, most of the previous studies were not designed to specifically address the questions of what the differences are in the cancer signaling pathways between the HBV and HCV infected HCC and how the signaling differences are regulated. With the large-scale and multi-genomic data sets from the Cancer Genome Atlas (TCGA) [26], we have performed a genome-scale profiling comparison between HBV and HCV infected HCC at gene expression and methylation level. Our results showed a substantial difference of hepatocarcinogenesis between the HBV and HCV infected HCC and the results improve our understanding of the molecular landscape of HCC.

## Methods

### Data source

TCGA-Liver Hepatocellular Carcinoma (HCC) cohort with publicly available data (https://www.cbioportal.org/study/summary?id=lihc_tcga_pan_can_atlas_2018

;https://portal.gdc.cancer.gov/projects/TCGA-LIHC) was used for this study.

From this cohort, 87 HCC cases with gene expression dataset, epigenetic data, and copy number alteration data were selected containing 60 cases of HBV infected HCC, 18 cases of HCV infected HCC, and 9 cases of no virus infection. Thirty-four para-cancerous tissues were used as control including 25 cases with HBV infection and 9 cases without virus infection.

### Gene expression analysis

The gene expression data was obtained as raw count values from TCGA public level 3 transcription profiles. R packages (edgeR) were used for transcriptional profiling and the differential expressing assessment between HBV and HCV infected samples. *P*-values were corrected for multiple testing by computing q-values (false discovery rates). Then the significantly differentially expressed genes (DEGs, *P* < 0.05 and Fold change value larger than1) were selected out for the next step analysis.

### DNA methylation analysis

The DNA methylation data were obtained as beta values from TCGA public level 3 methylation profiles. R packages (Minfi) were used for the global and regional CpG-island methylation profiling. Individual samples and CpG sites with the high missing rate (> 5%) were excluded. Differentially methylated region (DMR) with *P*-value< 0.01 and differentially methylated position (DMP) with P-value< 0.01 were shown by circos.

### Copy number variants (CNV) analysis

The copy number variation (CNV) data were obtained as segmentation data from TCGA public level 3 data. GISTIC2 was used to analyze CNAs to delineate genome-wide focal DNA gain and loss.

### Gene function enrichment analysis

The Gene Ontology (GO) functional annotation of DEGs was accomplished using Biomart Database (http://plants.ensembl.org/biomart) and the KEGG (Kyoto Encyclopedia of Genes and Genomes) pathway annotation of DEGs was accomplished using BLASTP to align to KEGG database (www.kegg.jp) with a cutoff e-value of 10^− 5^. GO enrichment analysis provides all GO terms that significantly enriched in DEGs comparing with the background. The method first mapped all DEGs to GO terms in the database (http://www.geneontology.org/), calculating gene numbers for every term, then using the hypergeometric test to find significantly enriched GO terms in DEGs comparing to the genome background. The calculating formula is:

$$ P=1-\sum \limits_{i=0}^{m-1}\frac{\left(\begin{array}{c}M\\ {}i\end{array}\right)\left(\begin{array}{c}N-M\\ {}i-i\end{array}\right)}{\left(\begin{array}{c}N\\ {}n\end{array}\right)} $$

Where N is the number of all genes with GO annotation; n is the number of DEGs in N; M is the number of all genes annotated to the certain GO terms; m is the number of DEGs in M. The calculated *P*-value underwent through Bonferroni Correction, taking corrected *P*-value < = 0.05 as a threshold. GO terms fulfilling this condition were defined as significantly enriched GO terms in DEGs. Pathway enrichment analysis identifies significantly enriched metabolic pathways or signal transduction pathways in DEGs comparing with the whole genome background and the calculating formula was the same as that in GO enrichment analysis.

### Integrative analysis

Integrative analysis of RNA-seq and Methy-seq were performed to detect the *cis*-related correlations between CpG methylation and RNA expression. The core set of samples was used since all samples in this set had data available across the two platforms. For analysis involving the RNA-seq datasets, a log2-transformation was used to correct the skewness in the data. To calculate the relative distance to measure the relationship between gene expression and methylation, the fpkm of RNAs and the methylated beta value were normalized to [0, 1] by arbitrarily setting the highest number as 1 and all other numbers adjusted accordingly. The normalized values were used as X and Y axis. The distance was calculated as follows:

$$ \mathrm{Distance}=\sqrt{{\mathrm{X}}_2+{\mathrm{Y}}_2} $$

$$ \mathrm{Where}\ \mathrm{X}=\mathrm{normalized}\ \mathrm{methylation}\ \mathrm{value};\mathrm{Y}=\mathrm{normalized}\ \mathrm{expression}\ \mathrm{value}. $$

## Results

### Differential expression profile between HBV and HCV-infected HCC

**Fig. 1:**
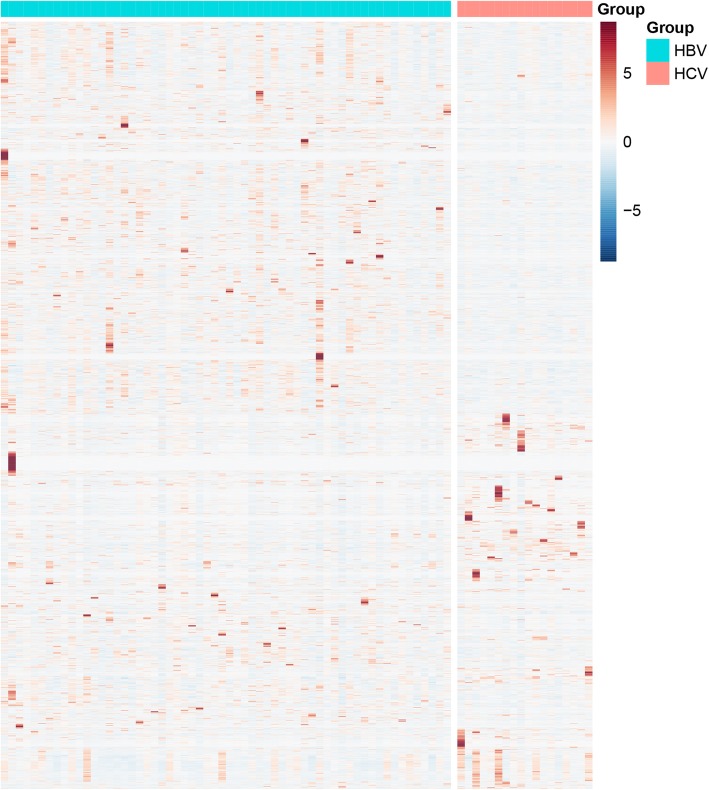
Heatmap of DEGs in the HCV-infected compared to HBV-infected HCCs The majority of differentially expressed genes were shown in the red boxed areas. The output thresholds were |logFC| > 1 and *P*-value< 0.05

The RNA-seq read counts data containing 60 HBV positive HCC and 18 HCV positive HCC were downloaded from TCGA. The clinical characteristics of all patients were shown in Additional file 1. The gene expression level was analyzed with the edgeR package using the software build-in normalization. The output thresholds were |logFC| > 1 and *P*-value< 0.05. Three thousand three hundred eighty-two differentially expressed transcripts (corresponding to 3152 genes) between HBV and HCV infected HCC were identified, as shown in Fig. 1. Among them, 1018 transcripts (954 genes) were up-regulated and 2346 transcripts (2198 genes) were down-regulated in the HCV samples compared with the HBV samples. The majority of differentially expressed genes were shown in the red boxed areas.

**Fig. 2:**
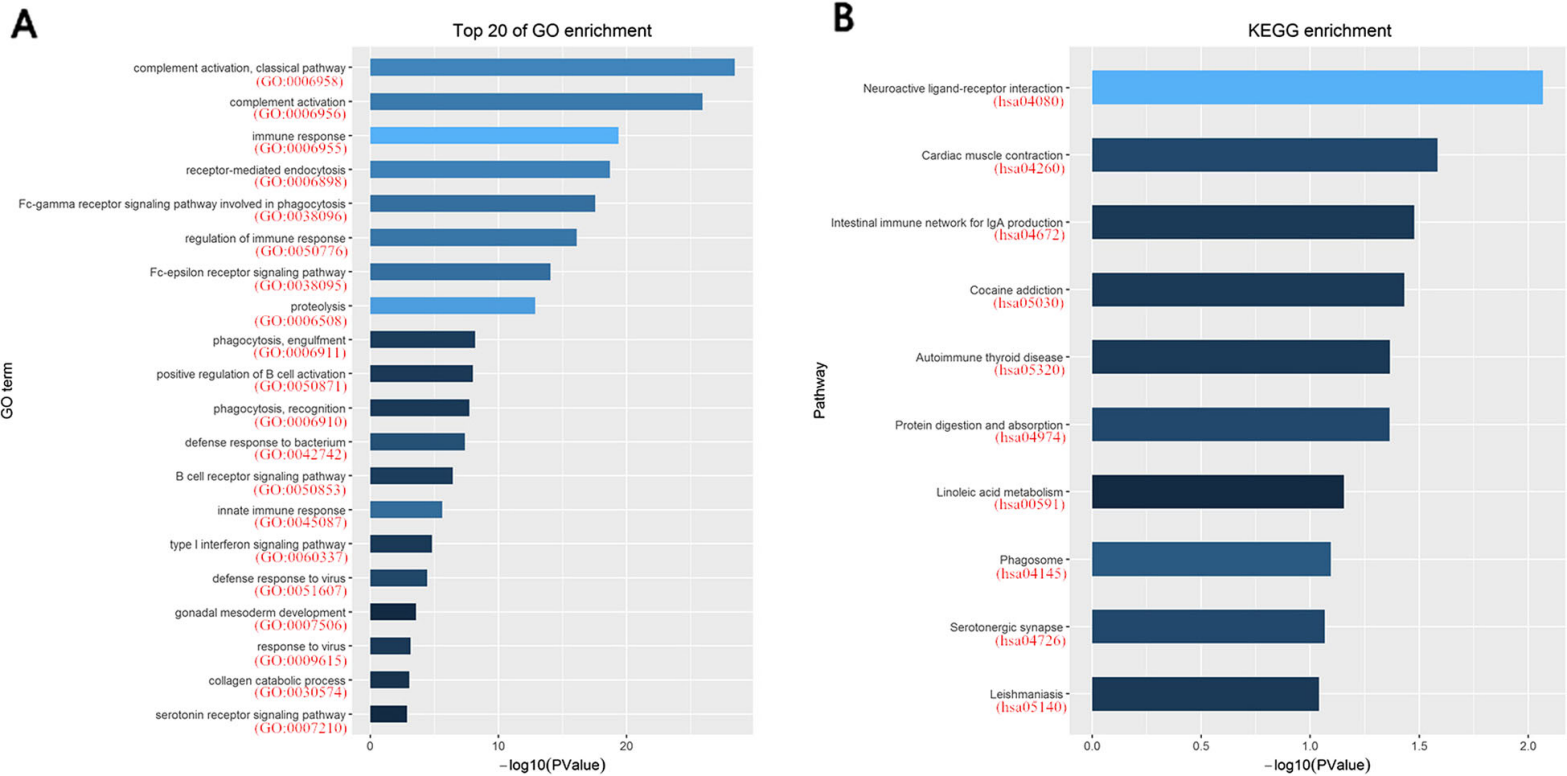
The GO and KEGG enrichment of up-regulated genes in the HCV-infected compared to HBV-infected HCCs. **a** Top 20 of GO enrichment of up-regulated genes in the HCV-infected compared to HBV-infected HCCs. **b** Top 20 of KEGG enrichment of up-regulated genes in the HCV-infected compared to HBV-infected HCCs. Functions and pathways involved in immunoregulation were shown in the red boxed areas

The 954 genes up-regulated in HCV-infected HCC were analyzed for GO and KEGG enrichment on the DAVID (version 6.8) online website (https://david.ncifcrf.gov/summary.jsp). The enriched results were shown in Fig. 2. According to the results of the GO Biological Process, those up-regulated genes in the HCV group were enriched in immune responses (Fig. 2a.) The gene lists were summarized in Additional file 2. It comprised: terms of complement activation; immune response; receptor-mediated endocytosis; phagocytosis; immune signaling pathway; defense response; etc. Immune system-related pathways were also listed on the KEGG enrichment (Fig. 2b, red boxes). The fact that up-regulated genes in HCV samples were enriched in immune system suggested HCV related HCC might have a markable difference upon the immune responses when compared with the HBV related HCC. The HCV specific down-regulated genes did not show significant enrichment in specific pathways (data not shown).

### Methylation profiles of HBV-infected and HCV-infected HCC

**Fig. 3:**
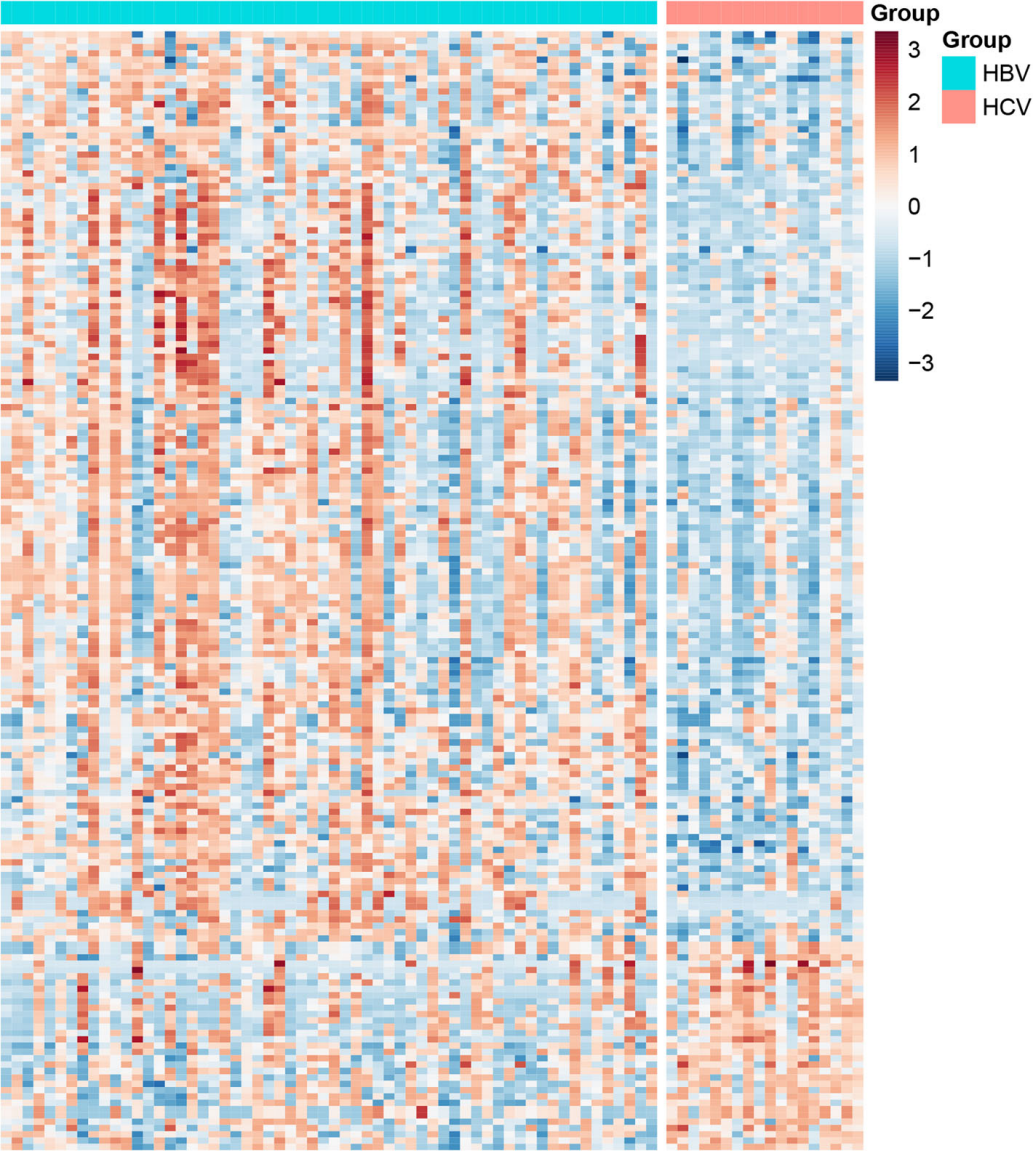
Heatmap of differential methylated genes in the HCV-infected compared to HBV-infected HCCs. The output thresholds were |delta beta value| > 0.15, *P* value< 0.01

**Fig. 4:**
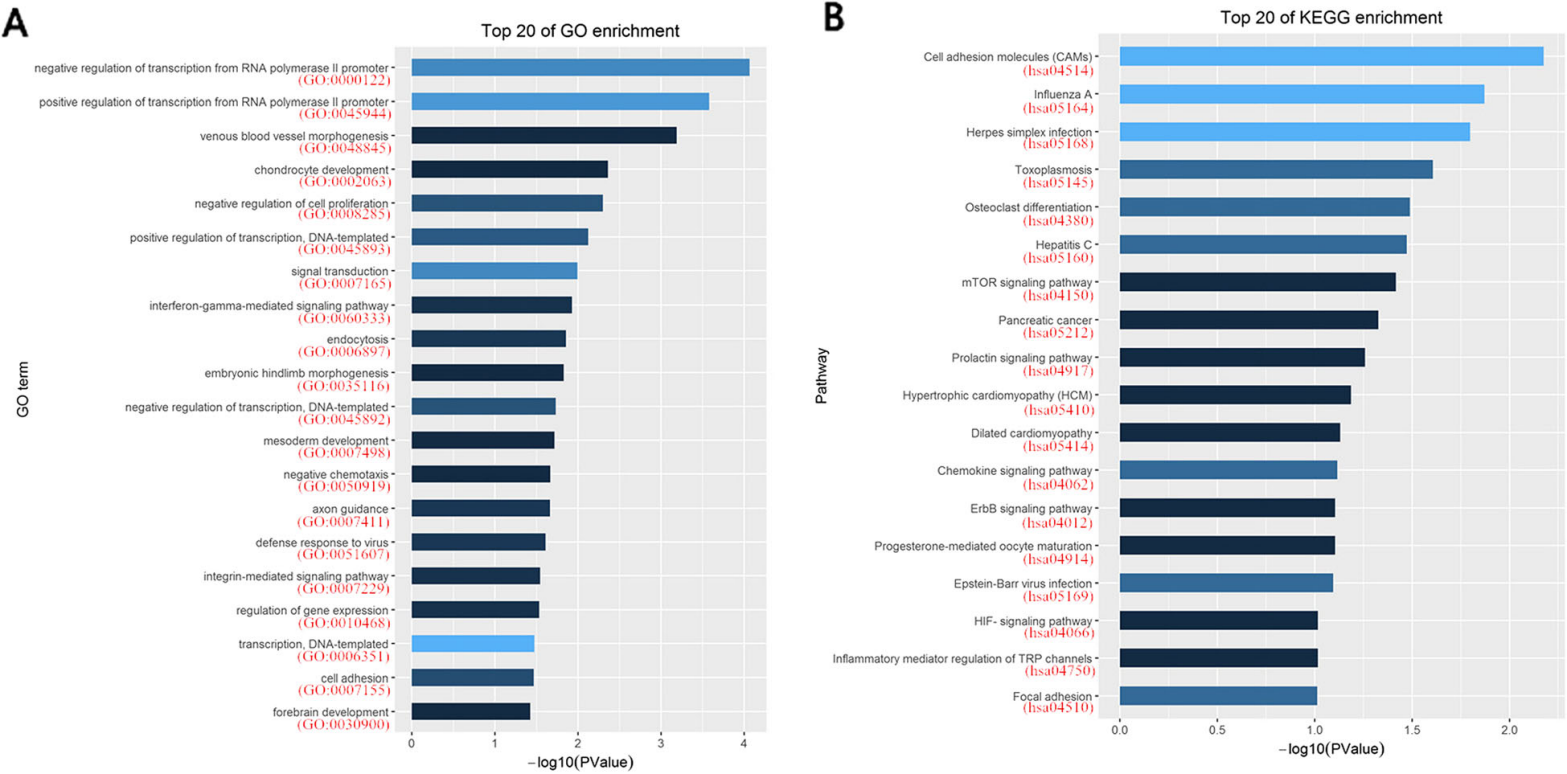
The GO and KEGG enrichment of hypomethylated genes in the HCV-infected compared to HBV-infected HCCs. **a** Top 20 of GO enrichment of hypomethylated genes in the HCV-infected compared to HBV-infected HCCs. **b** Top 20 of KEGG enrichment of hypomethylated genes in the HCV-infected compared to HBV-infected HCCs

The methylation data downloaded from TCGA was the Beta value of the CpG loci, and the methylation profiles of HBV and HCV samples were analyzed via MinFi software package in R software. When |delta beta value| > 0.15, *P* value< 0.01, 43 CpG islands involving 33 genes and 254 CpG islands involving 144 genes were hypermethylated or hypomethylated respectively in HCV samples, as shown in Fig. 3. Also, unlike expression profile enriched in the immune system, the related biological processes of hypomethylated genes were slightly enriched in transcriptional pathways in GO and were scattered without significant features in KEGG, (Fig. 4 and Additional file 3). Because of the limited number of hypermethylated genes, the enrichment in GO or KEGG were not successful.

### Comprehensive analysis of gene expression and methylation profiles

**Fig. 5:**
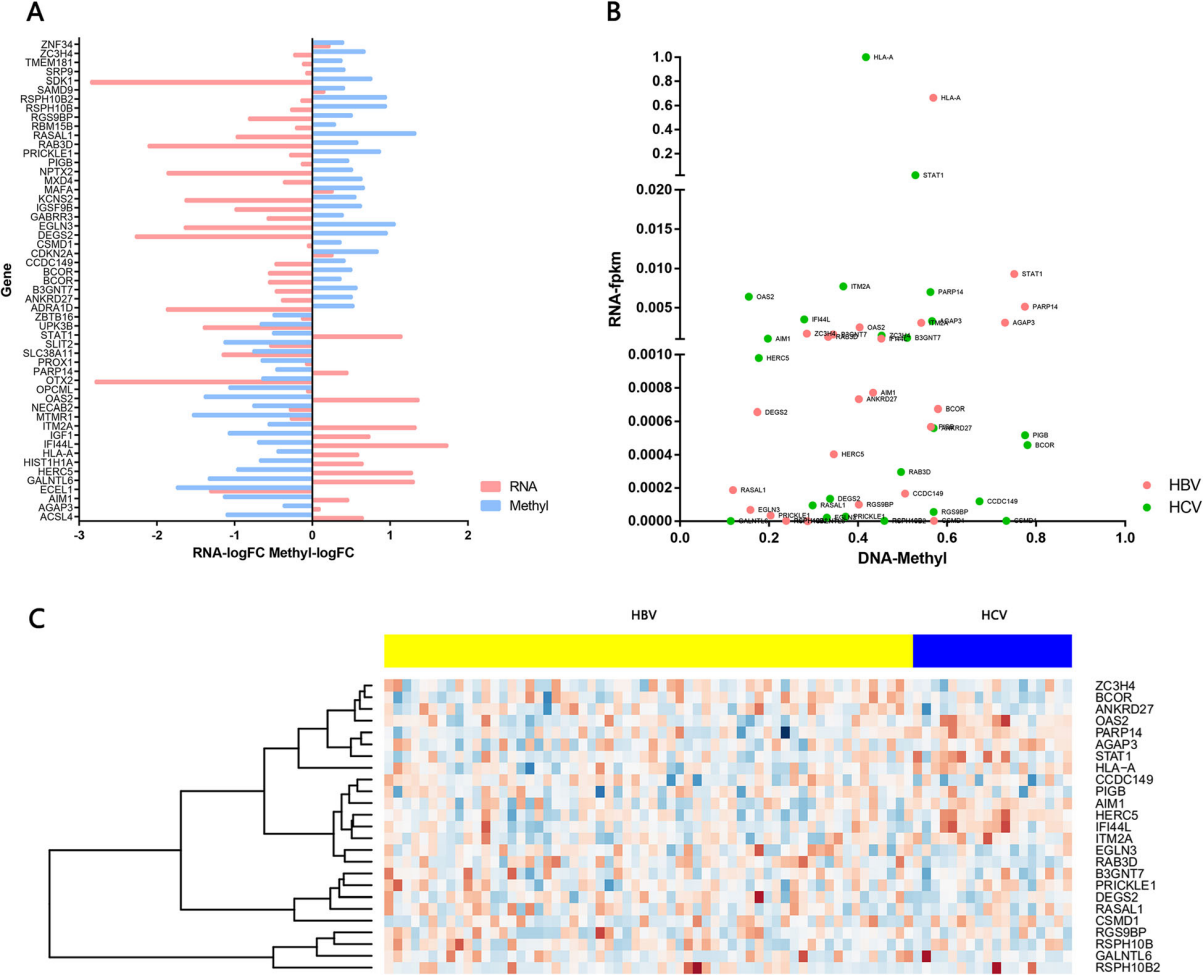
Association between RNA expression and Methylation of 25 cis-related genes. **a** Association between log (value) of RNA expression fold change and log value of methylation fold change of 25 cis-related genes in the HCV-infected compared to HBV-infected HCCs. **b** Scatter plot of RNA fpkm value and Methylation beta value of 25 cis-related genes in the HCV-infected and HBV-infected HCCs. **c** Heatmap of 25 cis-related genes in the HCV-infected and HBV-infected HCCs

According to the inverse correlation between methylation status and RNA expression, top 40 hyper or hypomethylated CpG islands (52 related genes) in HCV infected HCC were identified at |delta beta value| > 0.15 and *P*-value< 0.01 (Fig. 5a). Among the 52 genes, we used a relative distance to measure the expressional and methylation differences of the same gene between the HBV and HCV samples. For those 52 genes, the fpkm of RNAs and the methylated beta value were normalized to [0, 1]. The normalized values were used to map the gene distance between HBV and HCV samples, as shown in Fig. 5b and Additional file 4 (only the top 25 genes were displayed). HLA-A, STAT1, and OAS2genes differed the most between HBV and HCV infected HCC (the top 3 with the furthest distance). Compared with HBV infected HCC, those three genes were relatively highly expressed and low methylated in HCV infected HCC. The locations of 25 genes were also marked in circos together with DMR and DMP, as shown in Additional file 5.

For those top 25 genes on the distance map, the read counts from both HBV and HCV samples were processed via the DESeq2 package in R and the expression levels were normalized by the scale function with the heat map shown in Fig. 5c. There was a hot area in the HCV samples including the previously identified HLA-A, STAT1, and OAS2 genes as well as few additional genes (Fig. 5, red boxed area). These genes might represent the most differentially regulated genes between HBV and HCV infected HCC.

### Copy number variants (CNV) analysis

**Fig. 6:**
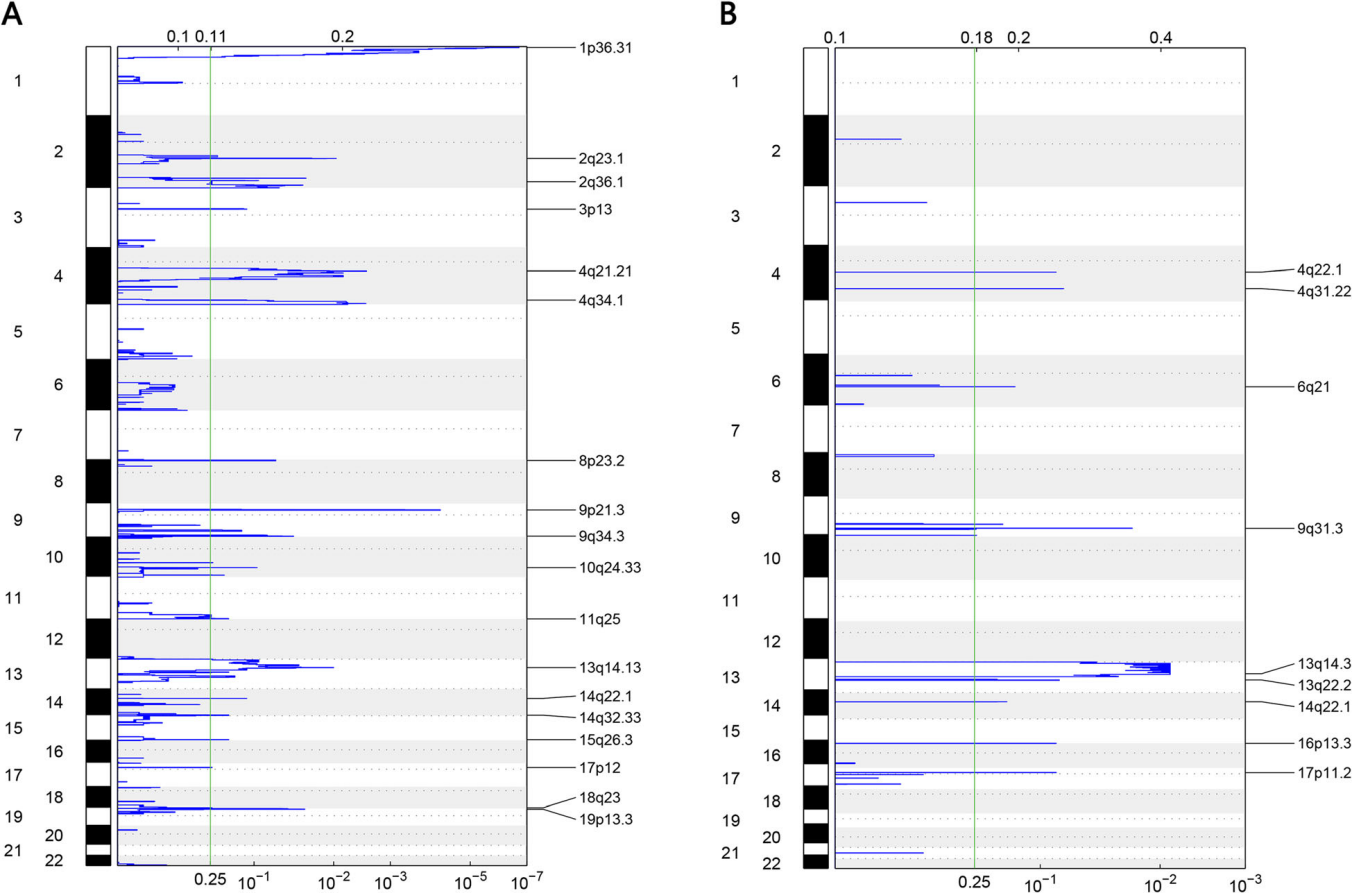
Genome-wide focal deletion peaks identified in HCC. **a** The deletion recurrent regions in the HBV-infected HCC samples. **b** The deletion recurrent regions in the HCV-infected HCC samples

The chromosome segment data of deletion or amplification from TCGA were analyzed with the gistic2.0 software for recurrent copy number variations (CNVs). The raw CNVs were shown in Additional file 6. In both sample sets, no amplification was discovered. The recurrent deletions were shown in Fig. 6. Notably, both HBV and HCV samples showed the same deletion region covered the area of chromesome13q14 (13q14.13 for HBV and 13q14.3 for HCV). The gene included in this region is RB1, a well-known tumor suppressor that was highly associated with liver cancer. This indicated a common mechanism for tumorigenesis in both HBV and HCV infected samples. Among the aforementioned 25 genes, only five genes including B3GNT7, DEGS2, CSMD1, GALNTL6, and HERC5 appeared in the CNV deletion regions and only in HBV samples. This result suggested the expression of other 20 genes including HLA-A, STAT1 and OAS2 may be only regulated by methylation, however, the five genes mentioned above B3GNT7, DEGS2, CSMD1, GALNTL6, and HERC5 might under the control of both methylation and CNV.

## Discussion

HBV and HCV infection are the two main risk factors responsible for HCC development in humans [7]. Previously, many researchers have investigated various mechanisms of tumorigenesis for HCC upon HBV and HCV infection [27, 28]. However, the differences between HBV and HCV related HCC, particularly the comprehensive genome-wide comparison between HBV/HCV infected HCC, have rarely been investigated before. Our knowledge of genetic and epigenetic changes in HCC upon viral infections is still limited. In this integrated analysis, we characterized genome-scale profiles of HBV and HCV infected HCC by comparing their gene expression pattern, methylation profiles, and copy number variations. We hope this study could improve our understanding of the epigenetic regulations in viral infected HCC and further benefit the clinical applications.

HBV and HCV belong to different types of virus (DNA vs. RNA) and the underlying mechanisms for hepatocarcinogenesis are different [29]. HBV contributes to the HCC development through the DNA integration into the host genome and therefore induces genomic instability and mutagenesis of diverse cancer-related genes [30, 31]. On the other hand, HCV infected cells will develop into HCC only two or more decades after the viral infection and the increased risk is restricted largely in the patients with cirrhosis or advanced fibrosis [32]. Therefore, there should be quite differently regulated genes in the cells of two types of HCC. In this study, we analyzed and characterized the genome-wide gene expression patterns, methylation profiles, and copy number variations of HBV/HCV infected HCC.

Compared with the HBV counterparts, the top up-regulated genes in HCV infected HCC were significantly clustered in the immune-related pathways. This might indicate a remarkably different response between two types of HCC upon the immunotherapy such as PD1/PD-L1 blockade, though this conjecture needs the validation from real clinical data. In the HCV infected HCC, the 52 hyper- or hypomethylated genes included several genes responsible for detoxification and immune response. Among them, the top three genes that showed the biggest difference between HBV and HCV samples at both expression and methylation levels were HLA-A, STAT1, and OAS2 genes. The HLA-A gene is closely related to the immune response pathway and the diversity of HLA-A is a protective shield against bacterial and viral invasion [33–35]. The STAT1 gene provides instructions for making a protein that is involved in multiple immune system functions and helps keep the immune system in balance by controlling the IL-17 pathway [36]. Multiple studies have demonstrated HCV infection elevated the IL-17 pathways and the relationship between IL-17 levels increases with the increasing liver disease progression and chronicity [37–40]. OAS2 gene, involved in the innate immune response to viral infection, was also found to be associated with the severity of liver disease in the HCV infected patients [41]. This analysis was based on TCGA database, in which most cases are from Caucasians and African Americans. Therefore, if a similar conclusion can be drawn in Asians remains to be determined, especially when the highly polymorphic HLA gene was considered. Nevertheless, previous researches including Asian population also found that the cell proliferation genes were predominantly expressed in HBV–HCC [42], while inflammatory phenotypes were enriched in HCV–HCC [43, 44]. Our comprehensive analysis of expression and methylation explained the phenotypes observed in previous studies at a molecular genetic level.

The patterns of recurrent CNVs also differed remarkably between HBV and HCV infected HCC while with a common deletion in RB1gene. RB1 is an important tumor suppressor gene and is closely related to the occurrence of human hepatocellular carcinoma [45]. The deletion of RB1 may be the common mechanism for the etiology of HBV and HCV infected HCC and similar results were observed previously [46].

## Conclusions

Using a bioinformatics approach, this study was designed to conduct the genome-wide comparison of HCC that were infected with HBV or HCV to understand the molecular similarity and difference between them. The results revealed the predisposing changes of gene expression in HCC, the top up-regulated genes in HCV group were significantly clustered in the immune-related pathways, and the top three genes were HLA-A, STAT1, and OAS2. Although the patient sample size is small and limited data available at the current stage, understanding different mechanisms of HBV and HCV pathogenesis will help elucidate the routes of virus-host interaction and further benefit anti-virus therapies.

## Supplementary information

**Additional file 1: Table S1.** Clinical Dataset of HCC.

**Additional file 2: Table S2.** GO and KEGG enrichment result of DEGs in HCV-infected compared with HBV-infected HCCs.

**Additional file 3: Table S3.** GO and KEGG enrichment result of Hypomethylated genes in HCV-infected compared with HBV-infected HCCs.

**Additional file 4: Table S4.** 25 cis-related genes RNA expression FPKM value. (PDF 235 kb)

**Additional file 5: Figure S1.** The Circos plot of differential methylation in HCV-infected compared with HBV-infected HCCs. The outer layer represents CpG islands heatmap. Red color represents CpG islands are hypermethylated. Blue color represents CpG islands are hypomethylated. The middle layer represents the scatter plot of a single CpG site. Each dot represents a significant different DNA methylation changes, with *p*-value associated at Y-axis. The inner layer of circular plot is top 25 genes with the largest betafc value in DNA methylation changes of CpG islands.

**Additional file 6: Figure S2.** Heatmap of copy number variation in HBV (A) and HCV (B).

## Data Availability

The datasets analysed during the current study are available in the cbioportal or TCGA repository (https://www.cbioportal.org/study/summary?id=lihc_tcga_pan_can_atlas_2018; https://portal.gdc.cancer.gov/projects/TCGA-LIHC), under the accession code: Hepatocellular Carcinoma (HCC).

## Abbreviations

CNV: Copy number variation
GO: Gene Ontology
HBV: Hepatitis B virus
HCC: Hepatocellular carcinoma
HCV: Hepatitis C virus
KEGG: Kyoto Encyclopedia of Genes and Genomes
NASH: Nonalcoholic steatohepatitis
TCGA: The Cancer Genome Atlas
