# Supplementary material for: A comprehensive genome-wide profiling comparison between HBV and HCV infected hepatocellular carcinoma
Source: BMC Med Genomics. 2019 Oct 28;12:147. doi: 10.1186/s12920-019-0580-x (PMC6819460; doi:10.1186/s12920-019-0580-x)
Supplement: Supplementary file 1 — Additional file 1: Table S1. Clinical Dataset of HCC. [file 12920_2019_580_MOESM1_ESM.pdf]

Table S1 Clinical Dataset of HCC

bcr_patient_barcode	viral_hepatitis_serology	tumor_tissue_site		bcr_patient_barcode	viral_hepatitis_serology	tumor_tissue_site
CDE_ID:2003301	CDE_ID:4395982	CDE_ID:3427536		CDE_ID:2003301	CDE_ID:4395982	CDE_ID:3427536
TCGA-2Y-A9GX	Hepatitis B Surface Antigen|HBV Surface Antibody|HBV Core Antibody	Liver		TCGA-2Y-A9H1	Hepatitis C Antibody	Liver
TCGA-5C-AAPD	HBV Surface Antibody	Liver		TCGA-2Y-A9HA	Hepatitis C Antibody	Liver
TCGA-BC-A10W	Hepatitis B Surface Antigen	Liver		TCGA-2Y-A9HB	Hepatitis C Virus RNA|HCV Genotype	Liver
TCGA-BD-A2L6	Hepatitis B Surface Antigen|HBV Surface Antibody|HBV Core Antibody	Liver		TCGA-FV-A4ZQ	Hepatitis C Virus RNA	Liver
TCGA-BD-A3ER	Hepatitis B Surface Antigen|HBV Surface Antibody|HBV Core Antibody	Liver		TCGA-GJ-A3OU	Hepatitis C Antibody	Liver
TCGA-DD-A113	Hepatitis B Surface Antigen|HBV Surface Antibody|HBV Core Antibody	Liver		TCGA-K7-A5RF	Hepatitis C Virus RNA	Liver
TCGA-DD-A115	Hepatitis B Surface Antigen|HBV Surface Antibody|HBV Core Antibody	Liver		TCGA-K7-A5RG	HCV Genotype	Liver
TCGA-DD-A116	Hepatitis B Surface Antigen|HBV Surface Antibody|HBV Core Antibody	Liver		TCGA-K7-A6G5	HCV Genotype	Liver
TCGA-DD-A118	Hepatitis B Surface Antigen|HBV Surface Antibody|HBV Core Antibody	Liver		TCGA-KR-A7K0	Hepatitis C Antibody	Liver
TCGA-DD-A119	Hepatitis B Surface Antigen|HBV Surface Antibody|HBV Core Antibody	Liver		TCGA-KR-A7K2	HCV Genotype	Liver
TCGA-DD-A11A	Hepatitis B Surface Antigen|HBV Core Antibody	Liver		TCGA-MI-A75E	HCV Genotype	Liver
TCGA-DD-A11B	Hepatitis B Surface Antigen|HBV Surface Antibody|HBV Core Antibody	Liver		TCGA-MI-A75G	HCV Genotype	Liver
TCGA-DD-A11C	Hepatitis B Surface Antigen|HBV Surface Antibody|HBV Core Antibody	Liver		TCGA-MI-A75H	HCV Genotype	Liver
TCGA-DD-A1EA	Hepatitis B Surface Antigen|HBV Surface Antibody|HBV Core Antibody|HBV DNA	Liver		TCGA-RG-A7D4	Hepatitis C Antibody	Liver
TCGA-DD-A1EB	Hepatitis B Surface Antigen|HBV Surface Antibody|HBV Core Antibody	Liver		TCGA-WX-AA44	Hepatitis C Antibody	Liver
TCGA-DD-A1ED	Hepatitis B Surface Antigen|HBV Surface Antibody|HBV Core Antibody	Liver		TCGA-WX-AA46	Hepatitis C Antibody	Liver
TCGA-DD-A1EF	Hepatitis B Surface Antigen|HBV Surface Antibody|HBV Core Antibody	Liver		TCGA-WX-AA47	Hepatitis C Antibody	Liver
TCGA-DD-A1EG	Hepatitis B Surface Antigen	Liver		TCGA-ZP-A9D2	Hepatitis C Virus RNA	Liver
TCGA-DD-A1EH	Hepatitis B Surface Antigen|HBV Surface Antibody|HBV Core Antibody|HBV DNA	Liver				
TCGA-DD-A1EI	Hepatitis B Surface Antigen|HBV DNA	Liver				
TCGA-DD-A1EJ	Hepatitis B Surface Antigen|HBV Surface Antibody|HBV Core Antibody	Liver				
TCGA-DD-A1EK	Hepatitis B Surface Antigen|HBV Surface Antibody|HBV Core Antibody	Liver				
TCGA-DD-A1EL	Hepatitis B Surface Antigen|HBV Surface Antibody|HBV Core Antibody|HBV DNA	Liver				
TCGA-DD-A39X	Hepatitis B Surface Antigen|HBV Surface Antibody	Liver				
TCGA-DD-A39Y	Hepatitis B Surface Antigen|HBV Surface Antibody|HBV Core Antibody	Liver				
TCGA-DD-A3A6	Hepatitis B Surface Antigen|HBV Surface Antibody|HBV Core Antibody	Liver				
TCGA-DD-A3A7	Hepatitis B Surface Antigen|HBV Surface Antibody|HBV Core Antibody	Liver				
TCGA-DD-A3A8	Hepatitis B Surface Antigen|HBV Surface Antibody|HBV Core Antibody	Liver				
TCGA-DD-A4NA	Hepatitis B Surface Antigen|HBV Surface Antibody	Liver				
TCGA-DD-A4NG	Hepatitis B Surface Antigen|HBV Surface Antibody|HBV Core Antibody	Liver				
TCGA-DD-A4NH	Hepatitis B Surface Antigen	Liver				
TCGA-DD-A4NI	Hepatitis B Surface Antigen|HBV Surface Antibody|HBV Core Antibody	Liver				
TCGA-DD-A4NJ	Hepatitis B Surface Antigen|HBV Surface Antibody|HBV Core Antibody	Liver				
TCGA-DD-A4NK	Hepatitis B Surface Antigen	Liver				
TCGA-DD-A4NL	Hepatitis B Surface Antigen|HBV Surface Antibody|HBV Core Antibody	Liver				
TCGA-DD-A4NP	Hepatitis B Surface Antigen|HBV Surface Antibody|HBV Core Antibody	Liver				
TCGA-DD-A4NR	Hepatitis B Surface Antigen|HBV Surface Antibody|HBV Core Antibody	Liver				
TCGA-DD-A4NS	Hepatitis B Surface Antigen|HBV Surface Antibody|HBV Core Antibody	Liver				
TCGA-DD-A4NV	Hepatitis B Surface Antigen|HBV Surface Antibody|HBV Core Antibody	Liver				
TCGA-DD-A73A	Hepatitis B Surface Antigen|HBV Surface Antibody|HBV Core Antibody	Liver				
TCGA-DD-A73B	Hepatitis B Surface Antigen	Liver				
TCGA-DD-A73C	Hepatitis B Surface Antigen|HBV Surface Antibody|HBV Core Antibody	Liver				
TCGA-DD-A73E	Hepatitis B Surface Antigen|HBV Surface Antibody	Liver				
TCGA-DD-A73G	Hepatitis B Surface Antigen|HBV Surface Antibody|HBV Core Antibody	Liver				
TCGA-DD-AA3A	Hepatitis B Surface Antigen|HBV Surface Antibody|HBV Core Antibody	Liver				
TCGA-ED-A459	Hepatitis B Surface Antigen	Liver				
TCGA-ED-A4XI	Hepatitis B Surface Antigen	Liver				
TCGA-ED-A5KG	Hepatitis B Surface Antigen	Liver				
TCGA-ED-A66X	Hepatitis B Surface Antigen	Liver				
TCGA-ED-A66Y	Hepatitis B Surface Antigen	Liver				
TCGA-ED-A7PX	Hepatitis B Surface Antigen	Liver				
TCGA-ED-A7PY	Hepatitis B Surface Antigen	Liver				
TCGA-ED-A7PZ	Hepatitis B Surface Antigen	Liver				
TCGA-ED-A7XP	Hepatitis B Surface Antigen	Liver				
TCGA-ED-A97K	Hepatitis B Surface Antigen	Liver				
TCGA-G3-AAV6	Hepatitis B Surface Antigen	Liver				
TCGA-G3-AAV7	Hepatitis B Surface Antigen|HBV Surface Antibody|HBV DNA	Liver				
TCGA-MI-A75C	HBV Surface Antibody	Liver				
TCGA-UB-A7ME	Hepatitis B Surface Antigen|HBV Core Antibody|HBV DNA	Liver				
TCGA-ZP-A9CZ	Hepatitis B Surface Antigen	Liver				
