# Supplementary material for: A comprehensive genome-wide profiling comparison between HBV and HCV infected hepatocellular carcinoma
Source: BMC Med Genomics. 2019 Oct 28;12:147. doi: 10.1186/s12920-019-0580-x (PMC6819460; doi:10.1186/s12920-019-0580-x)
Supplement: Supplementary file 2 — Additional file 2: Table S2. GO and KEGG enrichment result of DEGs in HCV-infected compared with HBV-infected HCCs. [file 12920_2019_580_MOESM2_ESM.pdf]

Table S2 GO and KEGG enrichment

Down-regulated Gene GO result

Category	Term	Count	%	PValue	List_Total	Pop Hits	Pop Total	Fold Enrichment	Bonferroni	Benjamini	FDR
GOTERM_BP_DIRECT	GO:0007156~homophilic cell adhesion via plasma membrane adhesion molecules	33	2.232746955	4.93435E-10	981	158	16792	3.575117098	1.52669E-06	1.52669E-06	8.91567E-07
GOTERM_BP_DIRECT	GO:0016266~O-glycan processing	18	1.217861976	3.2889E-08	981	60	16792	5.135168196	0.000101753	5.0878E-05	5.94258E-05
GOTERM_BP_DIRECT	GO:0048704~embryonic skeletal system morphogenesis	14	0.947225981	1.65028E-07	981	39	16792	6.144645704	0.000510466	0.000170184	0.000298182
GOTERM_BP_DIRECT	GO:0071805~potassium ion transmembrane transport	24	1.623815968	4.45806E-07	981	121	16792	3.395152526	0.001378373	0.000344771	0.000805506
GOTERM_BP_DIRECT	GO:0008544~epidermis development	19	1.285520974	1.54466E-06	981	85	16792	3.826203754	0.004767767	0.000955377	0.00279094
GOTERM_BP_DIRECT	GO:0009952~anterior/posterior pattern specification	18	1.217861976	2.85291E-06	981	80	16792	3.851376147	0.008788079	0.001470072	0.005154687
GOTERM_BP_DIRECT	GO:0006813~potassium ion transport	17	1.150202977	1.75209E-05	981	82	16792	3.548693469	0.052766914	0.007714388	0.031653048
GOTERM_BP_DIRECT	GO:0007409~axonogenesis	17	1.150202977	0.000165603	981	98	16792	2.969314943	0.400954552	0.062043988	0.298798748
GOTERM_BP_DIRECT	GO:0008344~adult locomotory behavior	12	0.811907984	0.0001677	981	52	16792	3.950129381	0.404829257	0.056025691	0.302576935
GOTERM_BP_DIRECT	GO:0034765~regulation of ion transmembrane transport	18	1.217861976	0.000233015	981	111	16792	2.775766592	0.513749627	0.069565094	0.420188805
GOTERM_BP_DIRECT	GO:1902476~chloride transmembrane transport	16	1.082543978	0.000300792	981	93	16792	2.944899324	0.60575774	0.081136082	0.542095681
GOTERM_BP_DIRECT	GO:0048791~calcium ion-regulated exocytosis of neurotransmitter	10	0.676589986	0.000327465	981	39	16792	4.389032646	0.636996037	0.080977917	0.5900321
GOTERM_BP_DIRECT	GO:0007399~nervous system development	33	2.232746955	0.000336971	981	287	16792	1.968182932	0.647520823	0.077079882	0.607111477
GOTERM_BP_DIRECT	GO:0035115~embryonic forelimb morphogenesis	9	0.608930988	0.000391497	981	32	16792	4.814220183	0.702257898	0.082899013	0.70502101
GOTERM_BP_DIRECT	GO:0030326~embryonic limb morphogenesis	10	0.676589986	0.000400913	981	40	16792	4.27930683	0.710810396	0.079383308	0.721919999
GOTERM_BP_DIRECT	GO:0007155~cell adhesion	46	3.112313938	0.000458726	981	459	16792	1.715451975	0.758194688	0.084904089	0.825615873
GOTERM_BP_DIRECT	GO:0034220~ion transmembrane transport	26	1.759133965	0.000556273	981	210	16792	2.119275763	0.821217648	0.09631079	1.000349161
GOTERM_BP_DIRECT	GO:0006836~neurotransmitter transport	8	0.541271989	0.000557171	981	26	16792	5.266839175	0.82171447	0.091352704	1.001958007
GOTERM_BP_DIRECT	GO:0007605~sensory perception of sound	19	1.285520974	0.000723317	981	133	16792	2.445318188	0.893408715	0.111152086	1.298896597
GOTERM_BP_DIRECT	GO:0017158~regulation of calcium ion-dependent exocytosis	9	0.608930988	0.000749715	981	35	16792	4.401572739	0.901774503	0.109546569	1.345998181
GOTERM_BP_DIRECT	GO:0007268~chemical synaptic transmission	28	1.894451962	0.000826428	981	240	16792	1.997009854	0.922542421	0.114684064	1.482754081
GOTERM_BP_DIRECT	GO:0006937~regulation of muscle contraction	6	0.405953992	0.000861259	981	14	16792	7.335954565	0.930462013	0.114122375	1.544787969
GOTERM_BP_DIRECT	GO:0001501~skeletal system development	19	1.285520974	0.001027763	981	137	16792	2.373922037	0.958478135	0.129186645	1.840825473
GOTERM_BP_DIRECT	GO:0007588~excretion	9	0.608930988	0.001108822	981	37	16792	4.163649888	0.967697104	0.133267542	1.984639074
GOTERM_BP_DIRECT	GO:0022617~extracellular matrix disassembly	13	0.879566982	0.001422704	981	76	16792	2.927946778	0.98778367	0.161549111	2.539652304
GOTERM_BP_DIRECT	GO:0007267~cell-cell signaling	28	1.894451962	0.001920202	981	254	16792	1.886938445	0.99738585	0.204451691	3.413259472
GOTERM_BP_DIRECT	GO:0007275~multicellular organism development	48	3.247631935	0.00201009	981	521	16792	1.577019024	0.998021622	0.205921733	3.570312099
GOTERM_BP_DIRECT	GO:0007411~axon guidance	20	1.353179973	0.002339112	981	159	16792	2.153110355	0.999286759	0.228003037	4.143124786
GOTERM_BP_DIRECT	GO:0006811~ion transport	17	1.150202977	0.002944866	981	127	16792	2.291282397	0.999891076	0.269955861	5.189318656
GOTERM_BP_DIRECT	GO:0007601~visual perception	23	1.556156969	0.003376294	981	201	16792	1.958687703	0.999971452	0.294464065	5.92784746
GOTERM_BP_DIRECT	GO:0006936~muscle contraction	15	1.01488498	0.003706334	981	107	16792	2.399611306	0.999989755	0.309682269	6.489147472
GOTERM_BP_DIRECT	GO:0070588~calcium ion transmembrane transport	16	1.082543978	0.003898968	981	119	16792	2.301475942	0.999994368	0.314576447	6.815295402
GOTERM_BP_DIRECT	GO:0042391~regulation of membrane potential	12	0.811907984	0.00400518	981	75	16792	2.738756371	0.99999595	0.313582992	6.994662415
GOTERM_BP_DIRECT	GO:0007193~adenylate cyclase-inhibiting G-protein coupled receptor signaling pathway	9	0.608930988	0.005395297	981	47	16792	3.277766933	0.999999946	0.388781795	9.312385315
GOTERM_BP_DIRECT	GO:0007565~female pregnancy	13	0.879566982	0.005450263	981	89	16792	2.500269159	0.999999955	0.383144831	9.402898839
GOTERM_BP_DIRECT	GO:0006486~protein glycosylation	15	1.01488498	0.006058379	981	113	16792	2.272198317	0.999999993	0.40682611	10.39861169
GOTERM_BP_DIRECT	GO:0030855~epithelial cell differentiation	11	0.744248985	0.007164716	981	70	16792	2.689850007	1	0.451891361	12.18364055
GOTERM_BP_DIRECT	GO:0010842~retina layer formation	6	0.405953992	0.007675729	981	22	16792	4.668334723	1	0.46600929	12.99674857
GOTERM_BP_DIRECT	GO:0045471~response to ethanol	14	0.947225981	0.008050469	981	105	16792	2.282296976	1	0.473369038	13.58849716
GOTERM_BP_DIRECT	GO:0008016~regulation of heart contraction	7	0.473612991	0.008152647	981	31	16792	3.865180362	1	0.469105459	13.74918476
GOTERM_BP_DIRECT	GO:0045617~negative regulation of keratinocyte differentiation	4	0.270635995	0.008892054	981	8	16792	8.55861366	1	0.490347401	14.90360921
GOTERM_BP_DIRECT	GO:0007269~neurotransmitter secretion	9	0.608930988	0.008902654	981	51	16792	3.020687174	1	0.482510273	14.92005226
GOTERM_BP_DIRECT	GO:0009888~tissue development	5	0.338294993	0.009375327	981	15	16792	5.70574244	1	0.492251812	15.65023176
GOTERM_BP_DIRECT	GO:0034587~piRNA metabolic process	5	0.338294993	0.009375327	981	15	16792	5.70574244	1	0.492251812	15.65023176
GOTERM_BP_DIRECT	GO:1901381~positive regulation of potassium ion transmembrane transport	5	0.338294993	0.009375327	981	15	16792	5.70574244	1	0.492251812	15.65023176
GOTERM_BP_DIRECT	GO:0032602~chemokine production	3	0.202976996	0.009811831	981	3	16792	17.11722732	1	0.50010502	16.31927564
GOTERM_BP_DIRECT	GO:0042472~inner ear morphogenesis	9	0.608930988	0.009997618	981	52	16792	2.962597036	1	0.498850586	16.60251432
GOTERM_BP_DIRECT	GO:0009954~proximal/distal pattern formation	6	0.405953992	0.011249266	981	24	16792	4.27930683	1	0.532765207	18.48722144
GOTERM_BP_DIRECT	GO:0045880~positive regulation of smoothened signaling pathway	6	0.405953992	0.011249266	981	24	16792	4.27930683	1	0.532765207	18.48722144
GOTERM_BP_DIRECT	GO:0036149~phosphatidylinositol acyl-chain remodeling	5	0.338294993	0.011932428	981	16	16792	5.349133537	1	0.546261078	19.49886634
GOTERM_BP_DIRECT	GO:0043588~skin development	7	0.473612991	0.012840338	981	34	16792	3.524135036	1	0.565268408	20.82497907
GOTERM_BP_DIRECT	GO:0009968~negative regulation of signal transduction	7	0.473612991	0.012840338	981	34	16792	3.524135036	1	0.565268408	20.82497907
GOTERM_BP_DIRECT	GO:0061337~cardiac conduction	8	0.541271989	0.014673882	981	45	16792	3.043062634	1	0.606791832	23.4404286
GOTERM_BP_DIRECT	GO:1903779~regulation of cardiac conduction	9	0.608930988	0.01539346	981	56	16792	2.750982962	1	0.617089862	24.44439132
GOTERM_BP_DIRECT	GO:0042755~eating behavior	6	0.405953992	0.015813534	981	26	16792	3.950129381	1	0.619785886	25.0247193
GOTERM_BP_DIRECT	GO:0008343~adult feeding behavior	4	0.270635995	0.01745428	981	10	16792	6.846890928	1	0.649256442	27.25130206
GOTERM_BP_DIRECT	GO:0045010~actin nucleation	4	0.270635995	0.01745428	981	10	16792	6.846890928	1	0.649256442	27.25130206
GOTERM_BP_DIRECT	GO:0036150~phosphatidylserine acyl-chain remodeling	5	0.338294993	0.018286383	981	18	16792	4.754785366	1	0.659517607	28.35649529
GOTERM_BP_DIRECT	GO:0043046~DNA methylation involved in gamete generation	5	0.338294993	0.018286383	981	18	16792	4.754785366	1	0.659517607	28.35649529
GOTERM_BP_DIRECT	GO:0060384~innervation	5	0.338294993	0.018286383	981	18	16792	4.754785366	1	0.659517607	28.35649529
GOTERM_BP_DIRECT	GO:0060349~bone morphogenesis	6	0.405953992	0.018498957	981	27	16792	3.803828293	1	0.656939354	28.63627921
GOTERM_BP_DIRECT	GO:0048863~stem cell differentiation	6	0.405953992	0.018498957	981	27	16792	3.803828293	1	0.656939354	28.63627921
GOTERM_BP_DIRECT	GO:0021759~globus pallidus development	3	0.202976996	0.018865438	981	4	16792	12.83792049	1	0.657473067	29.11621132
GOTERM_BP_DIRECT	GO:0097623~potassium ion export across plasma membrane	3	0.202976996	0.018865438	981	4	16792	12.83792049	1	0.657473067	29.11621132
GOTERM_BP_DIRECT	GO:0021615~glossopharyngeal nerve morphogenesis	3	0.202976996	0.018865438	981	4	16792	12.83792049	1	0.657473067	29.11621132
GOTERM_BP_DIRECT	GO:0035176~social behavior	8	0.541271989	0.020489485	981	48	16792	2.85287122	1	0.681394761	31.2065535
GOTERM_BP_DIRECT	GO:0031424~keratinization	8	0.541271989	0.020489485	981	48	16792	2.85287122	1	0.681394761	31.2065535
GOTERM_BP_DIRECT	GO:0006906~vesicle fusion	9	0.608930988	0.020636776	981	59	16792	2.611102472	1	0.67757948	31.39322602
GOTERM_BP_DIRECT	GO:2000463~positive regulation of excitatory postsynaptic potential	5	0.338294993	0.026402269	981	20	16792	4.27930683	1	0.760056343	38.33555962
GOTERM_BP_DIRECT	GO:0050896~response to stimulus	9	0.608930988	0.027035915	981	62	16792	2.484758804	1	0.762431625	39.05669363
GOTERM_BP_DIRECT	GO:0051965~positive regulation of synapse assembly	9	0.608930988	0.027035915	981	62	16792	2.484758804	1	0.762431625	39.05669363
GOTERM_BP_DIRECT	GO:0006821~chloride transport	7	0.473612991	0.027298414	981	40	16792	2.995514781	1	0.760034243	39.35309672
GOTERM_BP_DIRECT	GO:0016056~rhodopsin mediated signaling pathway	4	0.270635995	0.029329312	981	12	16792	5.70574244	1	0.779061913	41.60070473
GOTERM_BP_DIRECT	GO:0042446~hormone biosynthetic process	4	0.270635995	0.029329312	981	12	16792	5.70574244	1	0.779061913	41.60070473
GOTERM_BP_DIRECT	GO:0031581~hemidesmosome assembly	4	0.270635995	0.029329312	981	12	16792	5.70574244	1	0.779061913	41.60070473
GOTERM_BP_DIRECT	GO:0031133~regulation of axon diameter	3	0.202976996	0.03023368	981	5	16792	10.27033639	1	0.783904793	42.57604411
GOTERM_BP_DIRECT	GO:0007129~synapsis	5	0.338294993	0.031149808	981	21	16792	4.075530314	1	0.788627827	43.5483635
GOTERM_BP_DIRECT	GO:0030574~collagen catabolic process	9	0.608930988	0.031996177	981	64	16792	2.407110092	1	0.792391691	44.43280522
GOTERM_BP_DIRECT	GO:0048701~embryonic cranial skeleton morphogenesis	6	0.405953992	0.032168479	981	31	16792	3.313011739	1	0.789103827	44.61124698
GOTERM_BP_DIRECT	GO:0060021~palate development	10	0.676589986	0.032385688	981	76	16792	2.252266753	1	0.786331565	44.83542552
GOTERM_BP_DIRECT	GO:0007218~neuropeptide signaling pathway	12	0.811907984	0.033326808	981	101	16792	2.033729979	1	0.790960398	45.7968743
GOTERM_BP_DIRECT	GO:0008217~regulation of blood pressure	9	0.608930988	0.03469502	981	65	16792	2.370077629	1	0.799443434	47.16644553
GOTERM_BP_DIRECT	GO:0008284~positive regulation of cell proliferation	38	2.571041949	0.035769253	981	466	16792	1.395825404	1	0.804716395	48.21876836
GOTERM_BP_DIRECT	GO:0009953~dorsal/ventral pattern formation	6	0.405953992	0.036357148	981	32	16792	3.209480122	1	0.805422858	48.78625638
GOTERM_BP_DIRECT	GO:0051480~regulation of cytosolic calcium ion concentration	6	0.405953992	0.036357148	981	32	16792	3.209480122	1	0.805422858	48.78625638
GOTERM_BP_DIRECT	GO:0055074~calcium ion homeostasis	5	0.338294993	0.03636627	981	22	16792	3.890278936	1	0.800966834	48.79501567
GOTERM_BP_DIRECT	GO:0007214~gamma-aminobutyric acid signaling pathway	5	0.338294993	0.03636627	981	22	16792	3.890278936	1	0.800966834	48.79501567
GOTERM_BP_DIRECT	GO:0030818~negative regulation of cAMP biosynthetic process	4	0.270635995	0.036510721	981	13	16792	5.266839175	1	0.797761043	48.93352858
GOTERM_BP_DIRECT	GO:0030198~extracellular matrix organization	19	1.285520974	0.038120669	981	196	16792	1.659323056	1	0.80742913	50.45352806
GOTERM_BP_DIRECT	GO:0050796~regulation of insulin secretion	9	0.608930988	0.040545437	981	67	16792	2.299329043	1	0.822815339	52.66239284
GOTERM_BP_DIRECT	GO:0044267~cellular protein metabolic process	13	0.879566982	0.042666213	981	118	16792	1.88579623	1	0.834498635	54.51775366
GOTERM_BP_DIRECT	GO:0001503~ossification	10	0.676589986	0.043011429	981	80	16792	2.139653415	1	0.833004874	54.81318589
GOTERM_BP_DIRECT	GO:0042060~wound healing	10	0.676589986	0.043011429	981	80	16792	2.139653415	1	0.833004874	54.81318589
GOTERM_BP_DIRECT	GO:0045665~negative regulation of neuron differentiation	8	0.541271989	0.043366944	981	56	16792	2.445318188	1	0.831610695	55.11553693
GOTERM_BP_DIRECT	GO:0003416~endochondral bone growth	3	0.202976996	0.043616099	981	6	16792	8.55861366	1	0.829491437	55.32629256
GOTERM_BP_DIRECT	GO:0046881~positive regulation of follicle-stimulating hormone secretion	3	0.202976996	0.043616099	981	6	16792	8.55861366	1	0.829491437	55.32629256
GOTERM_BP_DIRECT	GO:0071774~response to fibroblast growth factor	3	0.202976996	0.043616099	981	6	16792	8.55861366	1	0.829491437	55.32629256
GOTERM_BP_DIRECT	GO:0001768~establishment of T cell polarity	3	0.202976996	0.043616099	981	6	16792	8.55861366	1	0.829491437	55.32629256
GOTERM_BP_DIRECT	GO:0001661~conditioned taste aversion	3	0.202976996	0.043616099	981	6	16792	8.55861366	1	0.829491437	55.32629256
GOTERM_BP_DIRECT	GO:2000147~positive regulation of cell motility	4	0.270635995	0.044503657	981	14	16792	4.890636377	1	0.831857041	56.06949126
GOTERM_BP_DIRECT	GO:0043524~negative regulation of neuron apoptotic process	14	0.947225981	0.044681129	981	132	16792	1.815463504	1	0.829298361	56.21668989
GOTERM_BP_DIRECT	GO:0008542~visual learning	7	0.473612991	0.045415408	981	45	16792	2.662679805	1	0.830581558	56.82077683
GOTERM_BP_DIRECT	GO:0045494~photoreceptor cell maintenance	6	0.405953992	0.045697676	981	34	16792	3.020687174	1	0.828794626	57.05089501
GOTERM_BP_DIRECT	GO:0036152~phosphatidylethanolamine acyl-chain remodeling	5	0.338294993	0.048215972	981	24	16792	3.566089025	1	0.841520139	59.05327621
GOTERM_BP_DIRECT	GO:0048666~neuron development	7	0.473612991	0.04975845	981	46	16792	2.604795462	1	0.84740056	60.23584981
GOTERM_BP_DIRECT	GO:1901379~regulation of potassium ion transmembrane transport	4	0.270635995	0.05328574	981	15	16792	4.564593952	1	0.863739916	62.82001971
GOTERM_BP_DIRECT	GO:0048485~sympathetic nervous system development	4	0.270635995	0.05328574	981	15	16792	4.564593952	1	0.863739916	62.82001971
GOTERM_BP_DIRECT	GO:0051216~cartilage development	8	0.541271989	0.055017475	981	59	16792	2.320979975	1	0.869434775	64.02986586
GOTERM_BP_DIRECT	GO:0014049~positive regulation of glutamate secretion	3	0.202976996	0.058739364	981	7	16792	7.335954565	1	0.883844626	66.50541268
GOTERM_BP_DIRECT	GO:1902259~regulation of delayed rectifier potassium channel activity	3	0.202976996	0.058739364	981	7	16792	7.335954565	1	0.883844626	66.50541268
GOTERM_BP_DIRECT	GO:0010642~negative regulation of platelet-derived growth factor receptor signaling pathway	3	0.202976996	0.058739364	981	7	16792	7.335954565	1	0.883844626	66.50541268
GOTERM_BP_DIRECT	GO:0019227~neuronal action potential propagation	3	0.202976996	0.058739364	981	7	16792	7.335954565	1	0.883844626	66.50541268
GOTERM_BP_DIRECT	GO:0021520~spinal cord motor neuron cell fate specification	3	0.202976996	0.058739364	981	7	16792	7.335954565	1	0.883844626	66.50541268
GOTERM_BP_DIRECT	GO:0009642~response to light intensity	3	0.202976996	0.058739364	981	7	16792	7.335954565	1	0.883844626	66.50541268
GOTERM_BP_DIRECT	GO:0042756~drinking behavior	3	0.202976996	0.058739364	981	7	16792	7.335954565	1	0.883844626	66.50541268
GOTERM_BP_DIRECT	GO:0030148~sphingolipid biosynthetic process	7	0.473612991	0.059184611	981	48	16792	2.496262317	1	0.88293172	66.79053899
GOTERM_BP_DIRECT	GO:0010862~positive regulation of pathway-restricted SMAD protein phosphorylation	7	0.473612991	0.059184611	981	48	16792	2.496262317	1	0.88293172	66.79053899
GOTERM_BP_DIRECT	GO:0051930~regulation of sensory perception of pain	5	0.338294993	0.061944471	981	26	16792	3.291774484	1	0.89171907	68.50738462
GOTERM_BP_DIRECT	GO:0006182~cGMP biosynthetic process	4	0.270635995	0.062828812	981	16	16792	4.27930683	1	0.892552234	69.03953408
GOTERM_BP_DIRECT	GO:0007416~synapse assembly	8	0.541271989	0.063769944	981	61	16792	2.244882271	1	0.893583812	69.59652193
GOTERM_BP_DIRECT	GO:0007154~cell communication	6	0.405953992	0.06828643	981	38	16792	2.702720103	1	0.907326737	72.14030601
GOTERM_BP_DIRECT	GO:0030049~muscle filament sliding	6	0.405953992	0.06828643	981	38	16792	2.702720103	1	0.907326737	72.14030601
GOTERM_BP_DIRECT	GO:0060395~SMAD protein signal transduction	8	0.541271989	0.068445184	981	62	16792	2.208674493	1	0.905463308	72.22595271
GOTERM_BP_DIRECT	GO:0007613~memory	8	0.541271989	0.068445184	981	62	16792	2.208674493	1	0.905463308	72.22595271
GOTERM_BP_DIRECT	GO:0036151~phosphatidylcholine acyl-chain remodeling	5	0.338294993	0.069500213	981	27	16792	3.169856911	1	0.906610161	72.78884736
GOTERM_BP_DIRECT	GO:0030154~cell differentiation	36	2.435723951	0.070442551	981	462	16792	1.333809921	1	0.90735818	73.28248997
GOTERM_BP_DIRECT	GO:0032331~negative regulation of chondrocyte differentiation	4	0.270635995	0.073100015	981	17	16792	4.027582899	1	0.913404158	74.6294313
GOTERM_BP_DIRECT	GO:0010466~negative regulation of peptidase activity	4	0.270635995	0.073100015	981	17	16792	4.027582899	1	0.913404158	74.6294313
GOTERM_BP_DIRECT	GO:0050908~detection of light stimulus involved in visual perception	4	0.270635995	0.073100015	981	17	16792	4.027582899	1	0.913404158	74.6294313
GOTERM_BP_DIRECT	GO:0008277~regulation of G-protein coupled receptor protein signaling pathway	6	0.405953992	0.074745252	981	39	16792	2.633419588	1	0.91608482	75.43089578
GOTERM_BP_DIRECT	GO:0002062~chondrocyte differentiation	6	0.405953992	0.074745252	981	39	16792	2.633419588	1	0.91608482	75.43089578
GOTERM_BP_DIRECT	GO:0051963~regulation of synapse assembly	3	0.202976996	0.075355134	981	8	16792	6.418960245	1	0.915709048	75.7218718
GOTERM_BP_DIRECT	GO:0014902~myotube differentiation	3	0.202976996	0.075355134	981	8	16792	6.418960245	1	0.915709048	75.7218718
GOTERM_BP_DIRECT	GO:0060346~bone trabecula formation	3	0.202976996	0.075355134	981	8	16792	6.418960245	1	0.915709048	75.7218718
GOTERM_BP_DIRECT	GO:0071711~basement membrane organization	3	0.202976996	0.075355134	981	8	16792	6.418960245	1	0.915709048	75.7218718
GOTERM_BP_DIRECT	GO:1904322~cellular response to forskolin	3	0.202976996	0.075355134	981	8	16792	6.418960245	1	0.915709048	75.7218718
GOTERM_BP_DIRECT	GO:0048672~positive regulation of collateral sprouting	3	0.202976996	0.075355134	981	8	16792	6.418960245	1	0.915709048	75.7218718
GOTERM_BP_DIRECT	GO:0035137~hindlimb morphogenesis	3	0.202976996	0.075355134	981	8	16792	6.418960245	1	0.915709048	75.7218718
GOTERM_BP_DIRECT	GO:0006002~fructose 6-phosphate metabolic process	3	0.202976996	0.075355134	981	8	16792	6.418960245	1	0.915709048	75.7218718
GOTERM_BP_DIRECT	GO:0060012~synaptic transmission, glycinergic	3	0.202976996	0.075355134	981	8	16792	6.418960245	1	0.915709048	75.7218718
GOTERM_BP_DIRECT	GO:0035116~embryonic hindlimb morphogenesis	5	0.338294993	0.077505569	981	28	16792	3.056647736	1	0.919642059	76.72208604
GOTERM_BP_DIRECT	GO:0019233~sensory perception of pain	7	0.473612991	0.081013214	981	52	16792	2.304242139	1	0.92675319	78.27049609
GOTERM_BP_DIRECT	GO:0043010~camera-type eye development	6	0.405953992	0.081523372	981	40	16792	2.567584098	1	0.926100602	78.48742296
GOTERM_BP_DIRECT	GO:0048468~cell development	6	0.405953992	0.081523372	981	40	16792	2.567584098	1	0.926100602	78.48742296
GOTERM_BP_DIRECT	GO:0009566~fertilization	6	0.405953992	0.081523372	981	40	16792	2.567584098	1	0.926100602	78.48742296
GOTERM_BP_DIRECT	GO:0001649~osteoblast differentiation	11	0.744248985	0.081873081	981	104	16792	1.810475965	1	0.925059625	78.63494121
GOTERM_BP_DIRECT	GO:0051384~response to glucocorticoid	8	0.541271989	0.083669354	981	65	16792	2.10673567	1	0.927540949	79.37772466
GOTERM_BP_DIRECT	GO:0045807~positive regulation of endocytosis	4	0.270635995	0.084062741	981	18	16792	3.803828293	1	0.926632226	79.53710643
GOTERM_BP_DIRECT	GO:0048557~embryonic digestive tract morphogenesis	4	0.270635995	0.084062741	981	18	16792	3.803828293	1	0.926632226	79.53710643
GOTERM_BP_DIRECT	GO:0036148~phosphatidylglycerol acyl-chain remodeling	4	0.270635995	0.084062741	981	18	16792	3.803828293	1	0.926632226	79.53710643
GOTERM_BP_DIRECT	GO:0048169~regulation of long-term neuronal synaptic plasticity	4	0.270635995	0.084062741	981	18	16792	3.803828293	1	0.926632226	79.53710643
GOTERM_BP_DIRECT	GO:0060037~pharyngeal system development	4	0.270635995	0.084062741	981	18	16792	3.803828293	1	0.926632226	79.53710643
GOTERM_BP_DIRECT	GO:0022400~regulation of rhodopsin mediated signaling pathway	5	0.338294993	0.085949568	981	29	16792	2.951246089	1	0.929218354	80.2855171
GOTERM_BP_DIRECT	GO:0048662~negative regulation of smooth muscle cell proliferation	5	0.338294993	0.085949568	981	29	16792	2.951246089	1	0.929218354	80.2855171
GOTERM_BP_DIRECT	GO:0001764~neuron migration	11	0.744248985	0.086020755	981	105	16792	1.793233338	1	0.927592563	80.31324058
GOTERM_BP_DIRECT	GO:0043408~regulation of MAPK cascade	6	0.405953992	0.088616282	981	41	16792	2.504960095	1	0.931651922	81.2992822
GOTERM_BP_DIRECT	GO:0007417~central nervous system development	12	0.811907984	0.091578516	981	120	16792	1.711722732	1	0.936172011	82.36759102
GOTERM_BP_DIRECT	GO:0006874~cellular calcium ion homeostasis	10	0.676589986	0.092638088	981	93	16792	1.840562077	1	0.936673247	82.73551798
GOTERM_BP_DIRECT	GO:0032330~regulation of chondrocyte differentiation	3	0.202976996	0.093238075	981	9	16792	5.70574244	1	0.936261509	82.94062966
GOTERM_BP_DIRECT	GO:0048103~somatic stem cell division	3	0.202976996	0.093238075	981	9	16792	5.70574244	1	0.936261509	82.94062966
GOTERM_BP_DIRECT	GO:0071435~potassium ion export	3	0.202976996	0.093238075	981	9	16792	5.70574244	1	0.936261509	82.94062966
GOTERM_BP_DIRECT	GO:0010459~negative regulation of heart rate	3	0.202976996	0.093238075	981	9	16792	5.70574244	1	0.936261509	82.94062966
GOTERM_BP_DIRECT	GO:0007589~body fluid secretion	3	0.202976996	0.093238075	981	9	16792	5.70574244	1	0.936261509	82.94062966
GOTERM_BP_DIRECT	GO:0031638~zymogen activation	3	0.202976996	0.093238075	981	9	16792	5.70574244	1	0.936261509	82.94062966
GOTERM_BP_DIRECT	GO:2000146~negative regulation of cell motility	3	0.202976996	0.093238075	981	9	16792	5.70574244	1	0.936261509	82.94062966
GOTERM_BP_DIRECT	GO:0007603~phototransduction, visible light	3	0.202976996	0.093238075	981	9	16792	5.70574244	1	0.936261509	82.94062966
GOTERM_BP_DIRECT	GO:0001778~plasma membrane repair	3	0.202976996	0.093238075	981	9	16792	5.70574244	1	0.936261509	82.94062966
GOTERM_BP_DIRECT	GO:0048706~embryonic skeletal system development	5	0.338294993	0.094819509	981	30	16792	2.85287122	1	0.937763951	83.47028511
GOTERM_BP_DIRECT	GO:0045879~negative regulation of smoothened signaling pathway	4	0.270635995	0.09567747	981	19	16792	3.603626804	1	0.937851226	83.75109538
GOTERM_BP_DIRECT	GO:0007520~myoblast fusion	4	0.270635995	0.09567747	981	19	16792	3.603626804	1	0.937851226	83.75109538
GOTERM_BP_DIRECT	GO:0010951~negative regulation of endopeptidase activity	12	0.811907984	0.09572873	981	121	16792	1.697576263	1	0.936403077	83.76772917
GOTERM_BP_DIRECT	GO:0001658~branching involved in ureteric bud morphogenesis	6	0.405953992	0.096018516	981	42	16792	2.445318188	1	0.935411619	83.86146278
GOTERM_BP_DIRECT	GO:0006814~sodium ion transport	9	0.608930988	0.099588876	981	81	16792	1.901914147	1	0.940535081	84.97515612

Down-regulated Gene KEGG result

Category	Term	Count	%	PValue	List_Total	Pop Hits	Pop Total	Fold Enrichment	Bonferroni	Benjamini	FDR
KEGG_PATHWAY	hsa04080:Neuroactive ligand-receptor interaction	42	2.841677943	6.62818E-10	360	277	6910	2.910348977	1.49797E-07	1.49797E-07	8.4738E-07
KEGG_PATHWAY	hsa05033:Nicotine addiction	12	0.811907984	3.7818E-06	360	40	6910	5.758333333	0.000854323	0.000427253	0.004834732
KEGG_PATHWAY	hsa04024:cAMP signaling pathway	26	1.759133965	2.97085E-05	360	198	6910	2.520482604	0.006691727	0.00223557	0.037974168
KEGG_PATHWAY	hsa00512:Mucin type O-Glycan biosynthesis	9	0.608930988	0.000134932	360	31	6910	5.572580645	0.030036394	0.007595192	0.172366967
KEGG_PATHWAY	hsa04512:ECM-receptor interaction	13	0.879566982	0.001672215	360	87	6910	2.868135377	0.31493179	0.072856916	2.116904893
KEGG_PATHWAY	hsa04020:Calcium signaling pathway	19	1.285520974	0.005246157	360	179	6910	2.037399131	0.695398932	0.179733006	6.503486924
KEGG_PATHWAY	hsa04974:Protein digestion and absorption	12	0.811907984	0.005585371	360	88	6910	2.617424242	0.71799546	0.165425411	6.910271732
KEGG_PATHWAY	hsa04723:Retrograde endocannabinoid signaling	13	0.879566982	0.005857345	360	101	6910	2.470572057	0.734901033	0.152916925	7.235244257
KEGG_PATHWAY	hsa00601:Glycosphingolipid biosynthesis - lacto and neolacto series	6	0.405953992	0.009805248	360	26	6910	4.429487179	0.892140064	0.219199617	11.83620776
KEGG_PATHWAY	hsa04744:Phototransduction	6	0.405953992	0.011533404	360	27	6910	4.265432099	0.927320593	0.230619566	13.78325023
KEGG_PATHWAY	hsa04726:Serotonergic synapse	13	0.879566982	0.012194099	360	111	6910	2.247997998	0.93751363	0.22281227	14.51709519
KEGG_PATHWAY	hsa04725:Cholinergic synapse	13	0.879566982	0.012194099	360	111	6910	2.247997998	0.93751363	0.22281227	14.51709519
KEGG_PATHWAY	hsa04727:GABAergic synapse	11	0.744248985	0.012211112	360	85	6910	2.483986928	0.93775638	0.206571304	14.53591538
KEGG_PATHWAY	hsa00591:Linoleic acid metabolism	6	0.405953992	0.015587381	360	29	6910	3.971264368	0.971289492	0.238994562	18.19618719
KEGG_PATHWAY	hsa00052:Galactose metabolism	6	0.405953992	0.01793064	360	30	6910	3.838888889	0.983245613	0.253289878	20.65100056
KEGG_PATHWAY	hsa05032:Morphine addiction	11	0.744248985	0.019083273	360	91	6910	2.32020757	0.987151166	0.251960989	21.83342448
KEGG_PATHWAY	hsa05217:Basal cell carcinoma	8	0.541271989	0.022772203	360	55	6910	2.791919192	0.994516555	0.277744523	25.50940491
KEGG_PATHWAY	hsa04713:Circadian entrainment	11	0.744248985	0.025021725	360	95	6910	2.22251462	0.996742713	0.286001307	27.67211346
KEGG_PATHWAY	hsa02010:ABC transporters	7	0.473612991	0.025044832	360	44	6910	3.053661616	0.996760113	0.272729275	27.69402508
KEGG_PATHWAY	hsa00565:Ether lipid metabolism	7	0.473612991	0.027679826	360	45	6910	2.985802469	0.998242477	0.283865175	30.152971
KEGG_PATHWAY	hsa05414:Dilated cardiomyopathy	10	0.676589986	0.029482639	360	84	6910	2.28505291	0.998844559	0.286921426	31.79067042
KEGG_PATHWAY	hsa04916:Melanogenesis	11	0.744248985	0.034182018	360	100	6910	2.111388889	0.999614224	0.31222796	35.89472397
KEGG_PATHWAY	hsa00592:alpha-Linolenic acid metabolism	5	0.338294993	0.038161241	360	25	6910	3.838888889	0.999848255	0.329476093	39.19056124
KEGG_PATHWAY	hsa04261:Adrenergic signaling in cardiomyocytes	14	0.947225981	0.039692053	360	146	6910	1.840563166	0.999894129	0.328317134	40.4163221
KEGG_PATHWAY	hsa05034:Alcoholism	16	1.082543978	0.041495541	360	177	6910	1.735091023	0.999930773	0.329068586	41.83118412
KEGG_PATHWAY	hsa05410:Hypertrophic cardiomyopathy (HCM)	9	0.608930988	0.04848764	360	78	6910	2.21474359	0.999986767	0.361932706	47.0288621
KEGG_PATHWAY	hsa04972:Pancreatic secretion	10	0.676589986	0.051515417	360	93	6910	2.063918757	0.999993561	0.368548364	49.14384371
KEGG_PATHWAY	hsa00910:Nitrogen metabolism	4	0.270635995	0.055010401	360	17	6910	4.516339869	0.999997204	0.377248008	51.48826489
KEGG_PATHWAY	hsa04728:Dopaminergic synapse	12	0.811907984	0.068327898	360	128	6910	1.799479167	0.999999887	0.43518108	59.53831679
KEGG_PATHWAY	hsa04510:Focal adhesion	17	1.150202977	0.069135045	360	206	6910	1.584007551	0.999999907	0.427822624	59.98417994
KEGG_PATHWAY	hsa04724:Glutamatergic synapse	11	0.744248985	0.071618133	360	114	6910	1.852095517	0.999999949	0.428687021	61.32758439
KEGG_PATHWAY	hsa04640:Hematopoietic cell lineage	9	0.608930988	0.07317848	360	85	6910	2.032352941	0.999999965	0.425366437	62.15036374
KEGG_PATHWAY	hsa04970:Salivary secretion	9	0.608930988	0.077230634	360	86	6910	2.00872093	0.999999987	0.433146932	64.21231868
KEGG_PATHWAY	hsa04971:Gastric acid secretion	8	0.541271989	0.083602047	360	73	6910	2.103500761	0.999999997	0.450036831	67.24600655
KEGG_PATHWAY	hsa05322:Systemic lupus erythematosus	12	0.811907984	0.088098744	360	134	6910	1.718905473	0.999999999	0.458285123	69.24237694
KEGG_PATHWAY	hsa04270:Vascular smooth muscle contraction	11	0.744248985	0.089567829	360	119	6910	1.774276377	0.999999999	0.454423188	69.8698822
KEGG_PATHWAY	hsa00500:Starch and sucrose metabolism	5	0.338294993	0.089624103	360	33	6910	2.908249158	0.999999999	0.4453782	69.89368285
KEGG_PATHWAY	hsa04730:Long-term depression	7	0.473612991	0.089798314	360	60	6910	2.239351852	0.999999999	0.437129794	69.96725382
KEGG_PATHWAY	hsa04390:Hippo signaling pathway	13	0.879566982	0.092969408	360	151	6910	1.65250184	1	0.440291329	71.27780879
KEGG_PATHWAY	hsa04260:Cardiac muscle contraction	8	0.541271989	0.093456381	360	75	6910	2.047407407	1	0.433665511	71.47433064
KEGG_PATHWAY	hsa00600:Sphingolipid metabolism	6	0.405953992	0.095069615	360	47	6910	2.45035461	1	0.431309837	72.11654453
KEGG_PATHWAY	hsa05205:Proteoglycans in cancer	16	1.082543978	0.096413631	360	200	6910	1.535555556	1	0.428131421	72.6413794
KEGG_PATHWAY	hsa04912:GnRH signaling pathway	9	0.608930988	0.09946878	360	91	6910	1.898351648	1	0.430937367	73.8007107

Up-regulated Gene GO result

Category	Term	Count	%	PValue	List_Total	Pop Hits	Pop Total	Fold Enrichment	Bonferroni	Benjamini	FDR
GOTERM_BP_DIRECT	GO:0006958~complement activation, classical pathway	31	5.290102389	3.7074E-29	297	99	16792	17.70404381	4.81221E-26	4.81221E-26	6.05218E-26
GOTERM_BP_DIRECT	GO:0006956~complement activation	28	4.778156997	1.17362E-26	297	87	16792	18.19636983	1.52335E-23	7.61676E-24	1.91588E-23
GOTERM_BP_DIRECT	GO:0006955~immune response	43	7.337883959	4.41093E-20	297	421	16792	5.774738677	5.72538E-17	1.90846E-17	7.20066E-17
GOTERM_BP_DIRECT	GO:0006898~receptor-mediated endocytosis	30	5.119453925	2.00966E-19	297	186	16792	9.119148474	2.60854E-16	6.52135E-17	3.28069E-16
GOTERM_BP_DIRECT	GO:0038096~Fc-gamma receptor signaling pathway involved in phagocytosis	25	4.266211604	2.85911E-18	297	127	16792	11.1296694	3.71112E-15	7.42225E-16	4.66738E-15
GOTERM_BP_DIRECT	GO:0050776~regulation of immune response	27	4.607508532	8.1859E-17	297	178	16792	8.576098059	1.44107E-13	2.39808E-14	1.77636E-13
GOTERM_BP_DIRECT	GO:0038095~Fc-epsilon receptor signaling pathway	25	4.266211604	9.07131E-15	297	178	16792	7.940831536	1.18168E-11	1.68809E-12	1.48659E-11
GOTERM_BP_DIRECT	GO:0006508~proteolysis	38	6.484641638	1.42631E-13	297	500	16792	4.296942761	1.85177E-10	2.31472E-11	2.32891E-10
GOTERM_BP_DIRECT	GO:0006911~phagocytosis, engulfment	10	1.706484642	6.87469E-09	297	35	16792	16.15392015	8.9233E-06	9.91482E-07	1.12226E-05
GOTERM_BP_DIRECT	GO:0050871~positive regulation of B cell activation	9	1.535836177	1.0059E-08	297	26	16792	19.57109557	1.30566E-05	1.30566E-06	1.6421E-05
GOTERM_BP_DIRECT	GO:0006910~phagocytosis, recognition	9	1.535836177	1.9408E-08	297	28	16792	18.17316017	2.51913E-05	2.29014E-06	3.16827E-05
GOTERM_BP_DIRECT	GO:0042742~defense response to bacterium	16	2.730375427	4.21255E-08	297	145	16792	6.23875537	5.46774E-05	4.55657E-06	6.87681E-05
GOTERM_BP_DIRECT	GO:0050853~B cell receptor signaling pathway	10	1.706484642	3.86067E-07	297	54	16792	10.47013343	0.00050099	3.85466E-05	0.000630237
GOTERM_BP_DIRECT	GO:0045087~innate immune response	24	4.09556314	2.45468E-06	297	430	16792	3.155649518	0.003181107	0.000227558	0.004007087
GOTERM_BP_DIRECT	GO:0060337~type I interferon signaling pathway	9	1.535836177	1.59433E-05	297	64	16792	7.950757576	0.020481849	0.001378683	0.026023521
GOTERM_BP_DIRECT	GO:0051607~defense response to virus	13	2.218430034	3.69151E-05	297	165	16792	4.454565861	0.046786799	0.002990313	0.06024532
GOTERM_BP_DIRECT	GO:0007506~gonadal mesoderm development	4	0.682593857	0.000284353	297	8	16792	28.26936027	0.308673264	0.02148022	0.463184456
GOTERM_BP_DIRECT	GO:0009615~response to virus	9	1.535836177	0.000734379	297	110	16792	4.625895317	0.614638646	0.051597532	1.192120374
GOTERM_BP_DIRECT	GO:0030574~collagen catabolic process	7	1.194539249	0.000906801	297	64	16792	6.183922559	0.691971128	0.060095434	1.470073834
GOTERM_BP_DIRECT	GO:0007210~serotonin receptor signaling pathway	4	0.682593857	0.00136018	297	13	16792	17.3965294	0.829106647	0.084546565	2.197445331
GOTERM_BP_DIRECT	GO:0042493~response to drug	14	2.389078498	0.002963267	297	304	16792	2.603756867	0.97876343	0.167589881	4.729113174
GOTERM_BP_DIRECT	GO:0071356~cellular response to tumor necrosis factor	8	1.365187713	0.00334212	297	110	16792	4.111906948	0.987032872	0.179232459	5.318361646
GOTERM_BP_DIRECT	GO:0070588~calcium ion transmembrane transport	8	1.365187713	0.005149501	297	119	16792	3.800922389	0.998770714	0.252754733	8.082674512
GOTERM_BP_DIRECT	GO:0007267~cell-cell signaling	12	2.04778157	0.005557311	297	254	16792	2.671120655	0.999278016	0.26021537	8.695837703
GOTERM_BP_DIRECT	GO:0006874~cellular calcium ion homeostasis	7	1.194539249	0.006037468	297	93	16792	4.255602621	0.999614275	0.269784401	9.412855808
GOTERM_BP_DIRECT	GO:0006954~inflammatory response	15	2.559726962	0.007471316	297	379	16792	2.237680232	0.999940776	0.312292319	11.52268719
GOTERM_BP_DIRECT	GO:0007268~chemical synaptic transmission	11	1.877133106	0.01035037	297	240	16792	2.591358025	0.999998636	0.393576974	15.6205241
GOTERM_BP_DIRECT	GO:0032148~activation of protein kinase B activity	4	0.682593857	0.010443522	297	26	16792	8.698264698	0.999998793	0.385334824	15.75008477
GOTERM_BP_DIRECT	GO:0022617~extracellular matrix disassembly	6	1.023890785	0.010982029	297	76	16792	4.4635832	0.999999404	0.389977506	16.49542152
GOTERM_BP_DIRECT	GO:0021680~cerebellar Purkinje cell layer development	3	0.511945392	0.015334407	297	11	16792	15.41965106	0.999999998	0.487578743	22.29627279
GOTERM_BP_DIRECT	GO:0002250~adaptive immune response	8	1.365187713	0.016029752	297	148	16792	3.056147056	0.999999999	0.49166717	23.18721099
GOTERM_BP_DIRECT	GO:0071346~cellular response to interferon-gamma	5	0.853242321	0.017987813	297	57	16792	4.959536889	1	0.521104403	25.64480585
GOTERM_BP_DIRECT	GO:0006935~chemotaxis	7	1.194539249	0.021060555	297	122	16792	3.244024949	1	0.567091706	29.35315879
GOTERM_BP_DIRECT	GO:0014059~regulation of dopamine secretion	3	0.511945392	0.021249111	297	13	16792	13.04739705	1	0.55955027	29.57496783
GOTERM_BP_DIRECT	GO:0050709~negative regulation of protein secretion	3	0.511945392	0.021249111	297	13	16792	13.04739705	1	0.55955027	29.57496783
GOTERM_BP_DIRECT	GO:0006968~cellular defense response	5	0.853242321	0.023738522	297	62	16792	4.559574237	1	0.58974552	32.44277561
GOTERM_BP_DIRECT	GO:0050910~detection of mechanical stimulus involved in sensory perception of sound	3	0.511945392	0.024505851	297	14	16792	12.11544012	1	0.591220183	33.30439605
GOTERM_BP_DIRECT	GO:0007586~digestion	5	0.853242321	0.025007377	297	63	16792	4.487200043	1	0.588703737	33.86196363
GOTERM_BP_DIRECT	GO:0042130~negative regulation of T cell proliferation	4	0.682593857	0.027095989	297	37	16792	6.112294112	1	0.608712375	36.13724834
GOTERM_BP_DIRECT	GO:0030593~neutrophil chemotaxis	5	0.853242321	0.029055913	297	66	16792	4.283236404	1	0.625201875	38.20533271
GOTERM_BP_DIRECT	GO:0014002~astrocyte development	3	0.511945392	0.031578884	297	16	16792	10.6010101	1	0.646991694	40.77505072
GOTERM_BP_DIRECT	GO:0045071~negative regulation of viral genome replication	4	0.682593857	0.033159636	297	40	16792	5.653872054	1	0.65616225	42.33360787
GOTERM_BP_DIRECT	GO:0007208~phospholipase C-activating serotonin receptor signaling pathway	2	0.341296928	0.034945188	297	2	16792	56.53872054	1	0.666893859	44.04775401
GOTERM_BP_DIRECT	GO:0051209~release of sequestered calcium ion into cytosol	4	0.682593857	0.035324584	297	41	16792	5.515972735	1	0.662300433	44.40576237
GOTERM_BP_DIRECT	GO:0048663~neuron fate commitment	3	0.511945392	0.035380127	297	17	16792	9.977421272	1	0.654452264	44.45799272
GOTERM_BP_DIRECT	GO:0070374~positive regulation of ERK1 and ERK2 cascade	8	1.365187713	0.035740602	297	175	16792	2.584627225	1	0.649989351	44.79585527
GOTERM_BP_DIRECT	GO:0060333~interferon-gamma-mediated signaling pathway	5	0.853242321	0.036620323	297	71	16792	3.981600038	1	0.651014679	45.61230913
GOTERM_BP_DIRECT	GO:0070098~chemokine-mediated signaling pathway	5	0.853242321	0.036620323	297	71	16792	3.981600038	1	0.651014679	45.61230913
GOTERM_BP_DIRECT	GO:0071347~cellular response to interleukin-1	5	0.853242321	0.036620323	297	71	16792	3.981600038	1	0.651014679	45.61230913
GOTERM_BP_DIRECT	GO:0008284~positive regulation of cell proliferation	15	2.559726962	0.037519673	297	466	16792	1.819915897	1	0.652197516	46.43525284
GOTERM_BP_DIRECT	GO:0002548~monocyte chemotaxis	4	0.682593857	0.037560746	297	42	16792	5.384640051	1	0.644870235	46.47255556
GOTERM_BP_DIRECT	GO:0051412~response to corticosterone	3	0.511945392	0.039348162	297	18	16792	9.42312009	1	0.654715268	48.07248825
GOTERM_BP_DIRECT	GO:0019731~antibacterial humoral response	4	0.682593857	0.042244748	297	44	16792	5.139883685	1	0.673887151	50.57027275
GOTERM_BP_DIRECT	GO:0042832~defense response to protozoan	3	0.511945392	0.043475972	297	19	16792	8.927166401	1	0.677379972	51.59743735
GOTERM_BP_DIRECT	GO:0071222~cellular response to lipopolysaccharide	6	1.023890785	0.049726837	297	113	16792	3.002055958	1	0.720060878	56.51039942
GOTERM_BP_DIRECT	GO:0007193~adenylate cyclase-inhibiting G-protein coupled receptor signaling pathway	4	0.682593857	0.049791289	297	47	16792	4.811806003	1	0.713730592	56.55852663
GOTERM_BP_DIRECT	GO:0050954~sensory perception of mechanical stimulus	2	0.341296928	0.051958662	297	3	16792	37.69248036	1	0.722670324	58.14812487
GOTERM_BP_DIRECT	GO:0071543~diphosphoinositol polyphosphate metabolic process	2	0.341296928	0.051958662	297	3	16792	37.69248036	1	0.722670324	58.14812487
GOTERM_BP_DIRECT	GO:0034241~positive regulation of macrophage fusion	2	0.341296928	0.051958662	297	3	16792	37.69248036	1	0.722670324	58.14812487
GOTERM_BP_DIRECT	GO:0002292~T cell differentiation involved in immune response	2	0.341296928	0.051958662	297	3	16792	37.69248036	1	0.722670324	58.14812487
GOTERM_BP_DIRECT	GO:0035902~response to immobilization stress	3	0.511945392	0.052183808	297	21	16792	8.076960077	1	0.71771401	58.31008334
GOTERM_BP_DIRECT	GO:0007626~locomotory behavior	5	0.853242321	0.061108625	297	84	16792	3.365400032	1	0.768120195	64.27625329
GOTERM_BP_DIRECT	GO:0030154~cell differentiation	14	2.389078498	0.066115004	297	462	16792	1.713294562	1	0.789368491	67.26200304
GOTERM_BP_DIRECT	GO:0050482~arachidonic acid secretion	3	0.511945392	0.066279327	297	24	16792	7.067340067	1	0.784485534	67.35591314
GOTERM_BP_DIRECT	GO:0007548~sex differentiation	3	0.511945392	0.066279327	297	24	16792	7.067340067	1	0.784485534	67.35591314
GOTERM_BP_DIRECT	GO:0006772~thiamine metabolic process	2	0.341296928	0.068673191	297	4	16792	28.26936027	1	0.790951863	68.69564649
GOTERM_BP_DIRECT	GO:1903237~negative regulation of leukocyte tethering or rolling	2	0.341296928	0.068673191	297	4	16792	28.26936027	1	0.790951863	68.69564649
GOTERM_BP_DIRECT	GO:0010513~positive regulation of phosphatidylinositol biosynthetic process	2	0.341296928	0.068673191	297	4	16792	28.26936027	1	0.790951863	68.69564649
GOTERM_BP_DIRECT	GO:1901907~diadenosine pentaphosphate catabolic process	2	0.341296928	0.068673191	297	4	16792	28.26936027	1	0.790951863	68.69564649
GOTERM_BP_DIRECT	GO:1901911~adenosine 5'-(hexahydrogen pentaphosphate) catabolic process	2	0.341296928	0.068673191	297	4	16792	28.26936027	1	0.790951863	68.69564649
GOTERM_BP_DIRECT	GO:0038094~Fc-gamma receptor signaling pathway	2	0.341296928	0.068673191	297	4	16792	28.26936027	1	0.790951863	68.69564649
GOTERM_BP_DIRECT	GO:1901909~diadenosine hexaphosphate catabolic process	2	0.341296928	0.068673191	297	4	16792	28.26936027	1	0.790951863	68.69564649
GOTERM_BP_DIRECT	GO:0016048~detection of temperature stimulus	2	0.341296928	0.068673191	297	4	16792	28.26936027	1	0.790951863	68.69564649
GOTERM_BP_DIRECT	GO:0050715~positive regulation of cytokine secretion	3	0.511945392	0.07122895	297	25	16792	6.784646465	1	0.797810984	70.06891812
GOTERM_BP_DIRECT	GO:0007275~multicellular organism development	15	2.559726962	0.078681784	297	521	16792	1.627794257	1	0.825142526	73.75766302
GOTERM_BP_DIRECT	GO:0009653~anatomical structure morphogenesis	5	0.853242321	0.079573183	297	92	16792	3.072756551	1	0.82376271	74.16908798
GOTERM_BP_DIRECT	GO:0035987~endodermal cell differentiation	3	0.511945392	0.081470095	297	27	16792	6.28208006	1	0.826377971	75.02453869
GOTERM_BP_DIRECT	GO:0050832~defense response to fungus	3	0.511945392	0.081470095	297	27	16792	6.28208006	1	0.826377971	75.02453869
GOTERM_BP_DIRECT	GO:0048247~lymphocyte chemotaxis	3	0.511945392	0.086750597	297	28	16792	6.057720058	1	0.841253667	77.26796408
GOTERM_BP_DIRECT	GO:0000060~protein import into nucleus, translocation	3	0.511945392	0.086750597	297	28	16792	6.057720058	1	0.841253667	77.26796408
GOTERM_BP_DIRECT	GO:0001662~behavioral fear response	3	0.511945392	0.092130637	297	29	16792	5.848833159	1	0.854869464	79.35813841
GOTERM_BP_DIRECT	GO:0045214~sarcomere organization	3	0.511945392	0.092130637	297	29	16792	5.848833159	1	0.854869464	79.35813841
GOTERM_BP_DIRECT	GO:0045776~negative regulation of blood pressure	3	0.511945392	0.097605093	297	30	16792	5.653872054	1	0.867321516	81.29883453

Up-regulated Gene KEGG result

Category	Term	Count	%	PValue	List_Total	Pop Hits	Pop Total	Fold Enrichment	Bonferroni	Benjamini	FDR
KEGG_PATHWAY	hsa04080:Neuroactive ligand-receptor interaction	11	1.877133106	0.008556689	105	277	6910	2.613374592	0.76797179	0.76797179	9.953083852
KEGG_PATHWAY	hsa04260:Cardiac muscle contraction	5	0.853242321	0.026050645	105	75	6910	4.387301587	0.988749195	0.893930186	27.53209975
KEGG_PATHWAY	hsa04672:Intestinal immune network for IgA production	4	0.682593857	0.033296456	105	47	6910	5.600810537	0.996838721	0.853235526	33.84223194
KEGG_PATHWAY	hsa05030:Cocaine addiction	4	0.682593857	0.037031783	105	49	6910	5.372206025	0.998363063	0.798855567	36.89430337
KEGG_PATHWAY	hsa05320:Autoimmune thyroid disease	4	0.682593857	0.04302032	105	52	6910	5.062271062	0.999433158	0.775770703	41.51879328
KEGG_PATHWAY	hsa04974:Protein digestion and absorption	5	0.853242321	0.043170357	105	88	6910	3.739177489	0.999448068	0.713594775	41.63055318
KEGG_PATHWAY	hsa00591:Linoleic acid metabolism	3	0.511945392	0.070026534	105	29	6910	6.807881773	0.999995635	0.828491771	58.75739776
KEGG_PATHWAY	hsa04145:Phagosome	6	1.023890785	0.080325522	105	153	6910	2.58076564	0.999999343	0.83125716	63.99668366
KEGG_PATHWAY	hsa04726:Serotonergic synapse	5	0.853242321	0.085777338	105	111	6910	2.964392964	0.999999761	0.816212815	66.5157415
KEGG_PATHWAY	hsa05140:Leishmaniasis	4	0.682593857	0.090989541	105	71	6910	3.707578806	0.999999909	0.80245276	68.77177837
