# Supplementary material for: A comprehensive genome-wide profiling comparison between HBV and HCV infected hepatocellular carcinoma
Source: BMC Med Genomics. 2019 Oct 28;12:147. doi: 10.1186/s12920-019-0580-x (PMC6819460; doi:10.1186/s12920-019-0580-x)
Supplement: Supplementary file 3 — Additional file 3: Table S3. GO and KEGG enrichment result of Hypomethylated genes in HCV-infected compared with HBV-infected HCCs. [file 12920_2019_580_MOESM3_ESM.pdf]

Table S3 methylation

Hypomethylated genes GO result											
Category	Term	Count	%	PValue	List_Total	Pop Hits	Pop Total	Fold Enrichment	Bonferroni	Benjamini	FDR
GOTERM_BP_DIRECT	GO:0000122~negative regulation of transcription from RNA polymerase II promoter	16	12.6984127	8.62879E-05	113	720	16792	3.302261554	0.06984001	0.06984001	0.133197359
GOTERM_BP_DIRECT	GO:0045944~positive regulation of transcription from RNA polymerase II promoter	18	14.28571429	0.000263196	113	981	16792	2.72663798	0.198163303	0.104546653	0.405760545
GOTERM_BP_DIRECT	GO:0048845~venous blood vessel morphogenesis	3	2.380952381	0.000649913	113	6	16792	74.30088496	0.420421389	0.166246922	0.999157078
GOTERM_BP_DIRECT	GO:0002063~chondrocyte development	3	2.380952381	0.004374348	113	15	16792	29.72035398	0.974729776	0.601294457	6.547270026
GOTERM_BP_DIRECT	GO:0008285~negative regulation of cell proliferation	9	7.142857143	0.005033168	113	396	16792	3.377312953	0.98549832	0.571171476	7.49788462
GOTERM_BP_DIRECT	GO:0045893~positive regulation of transcription, DNA-templated	10	7.936507937	0.007544275	113	515	16792	2.88547126	0.998259606	0.653176952	11.03887757
GOTERM_BP_DIRECT	GO:0007165~signal transduction	16	12.6984127	0.010172314	113	1161	16792	2.047914142	0.999811851	0.706379753	14.60872723
GOTERM_BP_DIRECT	GO:0060333~interferon-gamma-mediated signaling pathway	4	3.174603175	0.011885088	113	71	16792	8.371930699	0.999956	0.714614656	16.86270984
GOTERM_BP_DIRECT	GO:0006897~endocytosis	5	3.968253968	0.014033604	113	139	16792	5.345387407	0.999992915	0.732198774	19.61146171
GOTERM_BP_DIRECT	GO:0035116~embryonic hindlimb morphogenesis	3	2.380952381	0.014884776	113	28	16792	15.9216182	0.999996567	0.715840698	20.67673123
GOTERM_BP_DIRECT	GO:0045892~negative regulation of transcription, DNA-templated	9	7.142857143	0.01854927	113	499	16792	2.680192243	0.999999849	0.760233495	25.11397171
GOTERM_BP_DIRECT	GO:0007498~mesoderm development	3	2.380952381	0.019197087	113	32	16792	13.93141593	0.999999913	0.742116344	25.87382855
GOTERM_BP_DIRECT	GO:0050919~negative chemotaxis	3	2.380952381	0.02152653	113	34	16792	13.11192087	0.999999988	0.754500548	28.54697977
GOTERM_BP_DIRECT	GO:0007411~axon guidance	5	3.968253968	0.021852179	113	159	16792	4.673011632	0.999999991	0.73395513	28.91341229
GOTERM_BP_DIRECT	GO:0051607~defense response to virus	5	3.968253968	0.024626572	113	165	16792	4.503083937	0.999999999	0.75209034	31.96476257
GOTERM_BP_DIRECT	GO:0007229~integrin-mediated signaling pathway	4	3.174603175	0.028543681	113	99	16792	6.004111916	1	0.780967726	36.06483352
GOTERM_BP_DIRECT	GO:0010468~regulation of gene expression	4	3.174603175	0.029287305	113	100	16792	5.944070796	1	0.769384476	36.81660615
GOTERM_BP_DIRECT	GO:0006351~transcription, DNA-templated	21	16.66666667	0.033543637	113	1955	16792	1.596233846	1	0.796141799	40.9629215
GOTERM_BP_DIRECT	GO:0007155~cell adhesion	8	6.349206349	0.034029279	113	459	16792	2.590009062	1	0.783209492	41.41948273
GOTERM_BP_DIRECT	GO:0030900~forebrain development	3	2.380952381	0.037720762	113	46	16792	9.691419777	1	0.800711705	44.78350454
GOTERM_BP_DIRECT	GO:0010595~positive regulation of endothelial cell migration	3	2.380952381	0.037720762	113	46	16792	9.691419777	1	0.800711705	44.78350454
GOTERM_BP_DIRECT	GO:0060041~retina development in camera-type eye	3	2.380952381	0.057238323	113	58	16792	7.686298444	1	0.90509354	59.76428057
GOTERM_BP_DIRECT	GO:0050764~regulation of phagocytosis	2	1.587301587	0.058465295	113	9	16792	33.02261554	1	0.899489168	60.5655584
GOTERM_BP_DIRECT	GO:0045669~positive regulation of osteoblast differentiation	3	2.380952381	0.060771113	113	60	16792	7.430088496	1	0.898433724	62.03115637
GOTERM_BP_DIRECT	GO:0035136~forelimb morphogenesis	2	1.587301587	0.064748551	113	10	16792	29.72035398	1	0.903682556	64.44017838
GOTERM_BP_DIRECT	GO:0021895~cerebral cortex neuron differentiation	2	1.587301587	0.064748551	113	10	16792	29.72035398	1	0.903682556	64.44017838
GOTERM_BP_DIRECT	GO:0008360~regulation of cell shape	4	3.174603175	0.067151282	113	140	16792	4.245764855	1	0.902979276	65.82537599
GOTERM_BP_DIRECT	GO:0060337~type I interferon signaling pathway	3	2.380952381	0.068052876	113	64	16792	6.965707965	1	0.897132092	66.33200559
GOTERM_BP_DIRECT	GO:0001946~lymphangiogenesis	2	1.587301587	0.070990249	113	11	16792	27.01850362	1	0.898548241	67.93429693
GOTERM_BP_DIRECT	GO:0009950~dorsal/ventral axis specification	2	1.587301587	0.077190659	113	12	16792	24.76696165	1	0.909924754	71.08526119
GOTERM_BP_DIRECT	GO:0007264~small GTPase mediated signal transduction	5	3.968253968	0.082444904	113	246	16792	3.020361177	1	0.917032217	73.52623027
GOTERM_BP_DIRECT	GO:0060996~dendritic spine development	2	1.587301587	0.083350054	113	13	16792	22.86181076	1	0.912308326	73.92675445
GOTERM_BP_DIRECT	GO:0003016~respiratory system process	2	1.587301587	0.083350054	113	13	16792	22.86181076	1	0.912308326	73.92675445
GOTERM_BP_DIRECT	GO:0048712~negative regulation of astrocyte differentiation	2	1.587301587	0.083350054	113	13	16792	22.86181076	1	0.912308326	73.92675445
GOTERM_BP_DIRECT	GO:0043066~negative regulation of apoptotic process	7	5.555555556	0.083992261	113	455	16792	2.286181076	1	0.906928066	74.20748288
GOTERM_BP_DIRECT	GO:2000678~negative regulation of transcription regulatory region DNA binding	2	1.587301587	0.089468702	113	14	16792	21.22882427	1	0.914343052	76.48915549
GOTERM_BP_DIRECT	GO:0014912~negative regulation of smooth muscle cell migration	2	1.587301587	0.089468702	113	14	16792	21.22882427	1	0.914343052	76.48915549
GOTERM_BP_DIRECT	GO:0002009~morphogenesis of an epithelium	2	1.587301587	0.089468702	113	14	16792	21.22882427	1	0.914343052	76.48915549
GOTERM_BP_DIRECT	GO:0030324~lung development	3	2.380952381	0.091452513	113	76	16792	5.865859339	1	0.912698825	77.26803152
Hypomethylated gene KEGG result											
Category	Term	Count	%	PValue	List_Total	Pop Hits	Pop Total	Fold Enrichment	Bonferroni	Benjamini	FDR
KEGG_PATHWAY	hsa04514:Cell adhesion molecules (CAMs)	5	3.968253968	0.006670787	38	142	6910	6.40289103	0.60293399	0.60293399	7.575030279
KEGG_PATHWAY	hsa05164:Influenza A	5	3.968253968	0.013423762	38	174	6910	5.225347852	0.845107682	0.606436387	14.70524514
KEGG_PATHWAY	hsa05168:Herpes simplex infection	5	3.968253968	0.015900424	38	183	6910	4.968363532	0.890506548	0.521594626	17.19149156
KEGG_PATHWAY	hsa05145:Toxoplasmosis	4	3.174603175	0.024744974	38	118	6910	6.164139161	0.968499953	0.578713249	25.53886808
KEGG_PATHWAY	hsa04380:Osteoclast differentiation	4	3.174603175	0.032379157	38	131	6910	5.552430695	0.989350734	0.596852536	32.1169285
KEGG_PATHWAY	hsa05160:Hepatitis C	4	3.174603175	0.033651805	38	133	6910	5.468935497	0.9911194	0.544934706	33.16030041
KEGG_PATHWAY	hsa04150:mTOR signaling pathway	3	2.380952381	0.038223548	38	58	6910	9.405626134	0.995384367	0.536212808	36.7885473
KEGG_PATHWAY	hsa05212:Pancreatic cancer	3	2.380952381	0.046993274	38	65	6910	8.392712551	0.998696103	0.564081393	43.24870781
KEGG_PATHWAY	hsa04917:Prolactin signaling pathway	3	2.380952381	0.055039389	38	71	6910	7.683469236	0.999595339	0.580231194	48.63841447
KEGG_PATHWAY	hsa05410:Hypertrophic cardiomyopathy (HCM)	3	2.380952381	0.064992486	38	78	6910	6.993927126	0.999906139	0.604407186	54.65635682
KEGG_PATHWAY	hsa05414:Dilated cardiomyopathy	3	2.380952381	0.073969068	38	84	6910	6.494360902	0.999975208	0.618669409	59.52303082
KEGG_PATHWAY	hsa04062:Chemokine signaling pathway	4	3.174603175	0.076519284	38	186	6910	3.910582909	0.999983055	0.59966823	60.81566974
KEGG_PATHWAY	hsa04012:ErbB signaling pathway	3	2.380952381	0.078600438	38	87	6910	6.270417423	0.999987589	0.580625653	61.84244005
KEGG_PATHWAY	hsa04914:Progesterone-mediated oocyte maturation	3	2.380952381	0.078600438	38	87	6910	6.270417423	0.999987589	0.580625653	61.84244005
KEGG_PATHWAY	hsa05169:Epstein-Barr virus infection	4	3.174603175	0.080428254	38	190	6910	3.828254848	0.999990563	0.562419214	62.72385232
KEGG_PATHWAY	hsa04066:HIF-1 signaling pathway	3	2.380952381	0.096323433	38	98	6910	5.566595059	0.999999149	0.606160028	69.63960434
KEGG_PATHWAY	hsa04750:Inflammatory mediator regulation of TRP channels	3	2.380952381	0.096323433	38	98	6910	5.566595059	0.999999149	0.606160028	69.63960434
KEGG_PATHWAY	hsa04510:Focal adhesion	4	3.174603175	0.096918141	38	206	6910	3.530914665	0.999999223	0.584906059	69.87392356
