# Supplementary material for: A comprehensive genome-wide profiling comparison between HBV and HCV infected hepatocellular carcinoma
Source: BMC Med Genomics. 2019 Oct 28;12:147. doi: 10.1186/s12920-019-0580-x (PMC6819460; doi:10.1186/s12920-019-0580-x)
Supplement: Supplementary file 4 — Additional file 4: Table S4. 25 cis-related genes RNA expression FPKM value. (PDF 235 kb) [file 12920_2019_580_MOESM4_ESM.pdf]

Table S4 25 cis-realted genes RNA expression FPKM value

HBV 25 genes FPKM																									
Tags	PIGB	ITM2A	ANKRD27	RAB3D	OAS2	RASAL1	AIM1	STAT1	EGLN3	ZC3H4	AGAP3	IFI44L	HERC5	PRICKLE1	RSPH10B	B3GNT7	DEGS2	RSPH10B2	PARP14	GALNTL6	CCDC149	CSMD1	BCOR	RGS9BP	HLA-A
TCGA-ED-A97K-01	0.136989	0.562921	0.153518	0.211999	0.666971	0.160878	0.202319	1.101014	0.020931	0.403108	0.536782	0.301733	0.060673	0.013971	0.000608	1.467206	0.355681	0.000152	0.84714	3.90E-05	0.064515	0	0.114857	0.018954	74.1415
TCGA-DD-A3A6-01	0.014838	0.22692	0.026536	0.024368	0.209196	0.001402	0.040521	0.182509	0.000414	0.202862	0.483374	0.04352	0.040253	0.030605	0.000872	0.064167	0.005249	0	0.145095	7.47E-05	0.017331	0	0.012551	0.022664	186.2322
TCGA-DD-A116-01	0.072742	0.059066	0.041133	0.06993	0.248293	0.002695	0.029025	0.431949	0.001535	0.100906	0.507345	0.050244	0.031113	0.001324	0.000298	0.078919	0.020542	0	0.35163	1.64E-05	0.018342	0	0.030841	0.013937	117.8502
TCGA-BD-A3ER-01	0.062139	0.417318	0.050039	0.02527	0.227588	0.000581	0.05812	0.838734	0.006036	0.134295	0.313126	0.024417	0.019081	0.003305	0	0.068837	0.004095	0	0.571764	5.72E-06	0.020169	2.73E-05	0.03791	0	89.35747
TCGA-ED-A66X-01	0.113714	0.248914	0.202898	0.455232	0.41024	0.061302	0.036265	1.357269	0.004103	0.233635	0.330769	0.208902	0.024114	0.0127	0.000576	0.153736	0.02033	0.000287	0.617723	1.06E-05	0.021651	0	0.092395	0.004491	81.76048
TCGA-DD-A4NR-01	0.083689	0.623945	0.111315	0.02708	1.296659	0.013916	0.13827	3.444946	0.008313	0.234143	0.364202	0.245261	0.049227	0.001178	0.00045	0.309164	0.003936	0.000299	0.818205	0	0.025563	0.000867	0.048424	0.023369	101.8021
TCGA-DD-A4NP-01	0.055502	0.086936	0.040197	0.014864	0.148646	0.000332	0.034751	0.311349	0.000815	0.111058	0.23969	0.036774	0.048015	0.001285	9.17E-05	0.025767	0.006625	0	0.35296	6.73E-06	0.008559	0	0.021033	0.005721	69.07555
TCGA-DD-A11A-01	0.042472	0.084101	0.105064	0.017922	0.045861	0.000954	0.125858	0.662491	0.000806	0.131031	0.299679	0.016329	0.022433	0.000496	0.000791	0.048663	0.073127	0	0.458648	0.000585	0.009771	0.000104	0.042724	0.018496	36.35499
TCGA-ED-A7PY-01	0.052967	0.060101	0.033002	0.077574	0.034279	0.015693	0.00656	1.347437	0.004401	0.235453	0.157412	0.02728	0.005225	0.001213	0.002129	0.068372	0.021751	0	0.37035	8.69E-06	0.012021	0	0.046054	0.011069	19.07857
TCGA-DD-A4NS-01	0.073178	0.937356	0.089044	0.071763	0.253022	0.010678	0.133268	0.903321	0.003619	0.242547	0.438736	0.119755	0.043735	0.029576	0.000777	1.477553	0.14331	0.000155	0.62977	6.27E-05	0.018684	0.0005	0.086913	0.004844	56.207
TCGA-DD-A39X-01	0.032362	0.241698	0.076059	0.012796	0.158995	0.065646	0.020063	0.256058	0.001759	0.122959	0.378445	0.036578	0.031721	0.003278	0	0.022888	0.091915	0	0.348795	0.000262	0.017034	4.54E-05	0.026007	0.040421	94.0672
TCGA-ED-A5KG-01	0.181125	3.927934	0.143305	0.166917	3.144969	0.045203	0.412664	6.791388	0.009752	0.393597	0.318041	3.347718	0.449001	0.018731	0.000535	0.346725	0.05157	0	1.541442	1.97E-05	0.019202	7.04E-05	0.178009	0.00835	161.6836
TCGA-DD-A11C-01	0.068865	0.138747	0.085063	0.050446	0.242584	0.000413	0.219374	1.33565	0.001618	0.175393	0.258141	0.03264	0.065496	0.001289	0	0.046775	0.001876	0	0.684079	0	0.010633	0.001157	0.05831	0.008909	104.2745
TCGA-DD-A39Y-01	0.041243	0.103111	0.111503	0.004103	0.072078	0.002066	0.044672	0.384309	0.023237	0.145176	0.710028	0.038538	0.019366	0.000544	0	0.039292	0.002411	0	0.422243	0	0.033626	2.95E-05	0.033867	0.026716	61.36464
TCGA-DD-AA3A-01	0.1212	0.071607	0.10252	0.206914	0.090714	0.206947	0.011013	1.633041	0.00128	0.311944	0.41452	0.006742	0.003064	0.004018	0.001567	1.968722	0.016722	0.000586	0.53106	1.44E-05	0.030316	6.01E-05	0.118551	0.003055	155.5734
TCGA-DD-A11B-01	0.115299	0.251183	0.048598	0.118611	0.113475	0	0.30113	0.801149	0.001482	0.156941	0.158713	0.016857	0.023283	0.002279	0.000136	0.024026	0.029481	0	0.418209	0	0.010439	0.002218	0.068467	0	19.00701
TCGA-ED-A66Y-01	0.05748	0.042748	0.058417	0.389407	0.028665	0.203486	0.010145	0.531358	0.005795	0.302256	0.657617	0.005397	0.010677	0.00075	0.000157	0.005035	0.01905	0	0.298725	1.15E-05	0.018374	1.03E-05	0.178537	0	6.80292
TCGA-DD-A73B-01	0.047056	0.078737	0.041618	0.012321	0.064266	0	0.353751	0.559866	0.006966	0.123029	0.151285	0.003571	0.106791	8.87E-05	0.00013	0.026024	0.043429	0	0.896783	0.000257	0.010522	0	0.072193	0	62.17838
TCGA-DD-A73G-01	0.020747	0.019992	0.012891	0.294549	0.075667	0.011851	0.008359	0.252824	0.000344	0.084933	0.293652	0.015902	0.004454	0.001053	0.000147	0.335549	0.042378	0	0.282964	5.92E-05	0.018517	0	0.00815	0	34.05319
TCGA-DD-A1EF-01	0.065635	0.397036	0.066157	1.062064	0.116156	0.002771	0.068624	0.784102	0.00276	0.344268	0.261281	0.04877	0.010571	0.004538	0.000511	0.069681	0.02431	0.000255	0.863979	0	0.015355	0	0.178998	0	26.63624
TCGA-DD-A4NG-01	0.042092	0.136649	0.093301	0.008467	0.127964	0.000645	0.04437	0.406155	0.004626	0.140691	0.469781	0.024614	0.060143	0.000503	0.000134	0.040774	0.042133	0	0.355414	0	0.008386	1.17E-05	0.080139	0.016676	59.09038
TCGA-DD-A1EL-01	0.058906	0.097889	0.211557	0.114443	0.147629	0.013255	0.033935	1.43879	0.006908	0.196749	0.248013	0.088493	0.011919	0.000839	0.000392	0.045689	0.000644	9.79E-05	0.65501	2.52E-05	0.046936	0.000946	0.115689	0.019894	68.15352
TCGA-2Y-A9GX-01	0.081716	0.594176	0.087796	0.033002	0.370886	0.006163	0.192719	1.593208	0.004539	0.169517	0.406496	0.099668	0.125388	0.008443	0.000511	0.194167	0.008949	0	1.416152	1.88E-05	0.022344	3.73E-05	0.056548	0	114.3147
TCGA-DD-A4NL-01	0.044252	0.317516	0.029347	0.028136	0.104778	0.000819	0.096713	0.410087	0.001365	0.139982	0.150144	0.028327	0.022328	0.003016	0	0.064023	0.017829	0	0.283622	0	0.013637	0	0.033092	0	48.52261
TCGA-DD-A3A7-01	0.00824	0.01953	0.060436	0.22829	0.130717	0.047045	0.039603	0.503598	0.001403	0.084309	0.352122	0.005086	0.056015	0.000471	0.000197	0.018978	0.069215	0	0.198632	5.06E-05	0.018182	3.02E-05	0.016595	0.089096	173.5714
TCGA-MI-A75C-01	0.063309	0.019558	0.058289	0.006392	1.334179	0.024001	0.116487	0.978187	0.001426	0.133088	0.555396	0.133451	0.053293	0	0.000549	0.019831	0.011717	0	0.53587	0	0.015228	3.01E-06	0.028513	0.034245	64.84902
TCGA-BC-A10W-01	0.037962	0.124564	0.145802	0.192996	0.153976	0.019768	0.032842	1.226522	0.029092	0.25441	0.32709	0.015647	0.077885	0.000394	0	0.635274	0.043727	0	1.137923	0	0.050127	0	0.123591	0.005991	36.99775
TCGA-DD-A1ED-01	0.043549	0.344795	0.035361	0.034491	0.161558	0.000783	0.073296	0.526918	0.001669	0.137715	0.185005	0.071176	0.056286	0.001554	0	0.106032	0.003555	0.000108	0.340999	3.97E-06	0.012434	0	0.044794	0	52.33213
TCGA-ED-A7XP-01	0.098783	0.413033	0.150321	0.030326	0.283189	0.006918	0.020418	0.947704	0.001104	0.184633	0.398025	0.040821	0.049215	0.006042	0.00062	0.080893	0.043776	0	1.253516	1.71E-05	0.043204	3.40E-06	0.054155	0.019341	86.22372
TCGA-5C-AAPD-01	0.046368	0.65022	0.067234	0.074841	0.352745	0.002346	0.074829	1.744032	0.002936	0.112956	0.327754	0.084059	0.014981	0.002057	0.000354	0.109389	0.165606	0	0.425081	0	0.013626	0.001923	0.08011	0.00552	79.91856
TCGA-DD-A1EB-01	0.056888	0.207557	0.057994	0.02789	0.274777	0.000536	0.124779	2.206733	0.006633	0.23524	0.55463	0.043366	0.032325	0.001469	0.000296	0.022608	0.012167	0	0.674112	5.44E-06	0.009645	3.25E-06	0.064055	0.006935	108.7298
TCGA-DD-A119-01	0.021936	0.247742	0.032706	0.02881	0.420321	0.013778	0.008155	0.651948	0.00258	0.123683	0.298579	0.027573	0.065508	0.003477	0.000139	0.041381	0.015096	0.000139	0.28006	1.53E-05	0.012172	3.05E-06	0.028451	0.002173	100.2489
TCGA-DD-A4NK-01	0.060707	0.172439	0.039637	0.015977	0.092463	0.000585	0.038509	0.333114	0.000136	0.233371	0.43487	0.049011	0.06968	0.001383	0.000485	0.025979	0.002125	0	0.758713	2.27E-05	0.001804	1.42E-05	0.068779	0	20.0588
TCGA-DD-A1EH-01	0.131041	0.16958	0.100744	0.33341	0.104794	0.063172	0.018627	2.022816	0.00085	0.336322	0.305975	0.025582	0.066188	0.002944	0	0.115538	0.036412	0.000118	1.898775	4.33E-06	0.013671	1.03E-05	0.18167	0.00184	18.18755
TCGA-DD-A4NH-01	0.126008	0.058407	0.090336	0.489686	0.232958	0.022738	0.023112	1.164883	0.032784	0.202851	0.309125	0.019198	0.028191	0.00145	0.001571	0.345577	0.042817	0.000627	0.47734	4.04E-05	0.038694	3.10E-05	0.073122	0.026951	141.0018
TCGA-BD-A2L6-01	0.040534	0.293128	0.040907	0.011182	0.109958	0.000426	0.122003	0.58074	0.000414	0.195989	0.289463	0.023899	0.065299	0.001126	0.000353	0.013226	0.045597	0.000822	0.324454	0	0.001953	0.003159	0.052149	0.003671	108.8547
TCGA-UB-A7ME-01	0.047073	0.536132	0.125093	0.189136	0.410291	0.041502	0.032773	2.538609	0.005468	0.251054	0.304914	0.163415	0.01892	0.003466	0.000999	0.088261	0.108302	0.000285	0.376339	0	0.012031	0	0.103752	0.024494	87.27055
TCGA-DD-A73C-01	0.057392	0.582394	0.058498	0.080803	0.143956	0.00099	0.134675	0.621167	0.003864	0.161455	0.418502	0.074408	0.023795	0.006314	0.00041	0.02636	0.031897	0	0.389873	0	0.009541	6.00E-05	0.066391	0.004267	39.69026
TCGA-DD-A4NJ-01	0.114054	0.357391	0.085283	0.071937	0.333408	0.006324	0.086833	0.896448	0.000999	0.195976	0.408034	0.092706	0.090142	0.001467	0.000238	0.166467	0.019566	0.000238	1.269923	0	0.01551	1.04E-05	0.119201	0	87.94376
TCGA-DD-A4NI-01	0.054617	0.452407	0.025306	0.017771	0.241877	0.000411	0.141074	1.128875	0.001113	0.123765	0.264178	0.108659	0.044095	0.001669	0	0.112125	0.010067	0.000113	0.568179	8.34E-06	0.005063	0	0.038422	0.001771	33.74371
TCGA-G3-AAV6-01	0.02256	0.183492	0.091891	0.145073	0.049873	0.002901	0.02362	0.383677	0.003149	0.189714	0.431688	0.018627	0.00415	0.001439	0.00015	0.131539	0.013821	0	0.173318	5.52E-06	0.020616	0.000853	0.064964	0.021099	38.89409
TCGA-ED-A7PZ-01	0.060722	0.01544	0.102005	0.065878	0.019442	0.009302	0.01508	0.22811	0.006792	0.20777	0.217207	0.022668	0.029699	0.000325	0	0.025719	0.0413	0	0.364892	0.000222	0.010182	0.000998	0.03469	0.035159	34.0495
TCGA-DD-A4NA-01	0.130325	0.161559	0.280147	0.070565	0.126181	0.000662	0.039003	1.422058	0.125094	0.300398	0.277206	0.026689	0.013891	0.002909	0.000686	0.2622	2.271082	0	0.731676	8.06E-05	0.032411	0.000147	0.16631	0.068485	200.286
TCGA-DD-A73A-01	0.047176	0.42498	0.044748	0.020954	0.172025	0.002001	0.224802	0.742563	0.00034	0.224798	0.333531	0.029828	0.04988	0.001135	0	0.016282	0.003027	0.000184	0.823638	0	0.015344	4.04E-06	0.052924	0	51.00243
TCGA-DD-A4NV-01	0.0758	0.325603	0.062622	0.182387	0.150989	0.001287	0.192203	0.555553	0.000447	0.201719	0.701521	0.048204	0.019391	0.004149	0	0.049253	0.056933	0.000133	0.472472	3.43E-05	0.005221	0	0.055043	0.00208	31.78568
TCGA-DD-A1EJ-01	0.065417	0.06651	0.108279	0.319973	0.047874	0.010214	0.08116	0.904888	0.003177	0.143113	0.446916	0.013898	0.005165	0.000101	0	0.087753	0.00097	0.000196	0.052656	6.87E-05	0.008706	0.001325	0.19289	0.006143	34.72062
TCGA-ED-A7PX-01	0.071666	0.148322	0.12511	0.340929	0.284123	0.114961	0.016549	0.858104	0.005329	0.236087	0.264747	0.159084	0.019506	0.010303	0.000144	0.123868	0.202185	0	0.378007	0	0.043554	3.16E-05	0.079979	0.004498	233.8885
TCGA-DD-A118-01	0.032395	0.233691	0.051696	1.06377	0.115292	0.011574	0.009282	0.517287	0.002328	0.183091	0.151326	0.031303	0.004518	0.002327	0.000223	0.160346	0.039937	0.000111	0.619901	0.001209	0.020337	3.18E-05	0.066864	0.005221	24.25286
TCGA-ED-A459-01	0.086675	0.031967	0.160403	0.150494	0.363687	0.005333	0.0151	0.793587	0.028709	0.147105	0.591308	0.172509	0.032554	0.000655	0.000737	0.081651	0.011132	0	0.666163	0	0.051246	0	0.132518	0.006896	115.499
TCGA-DD-A1EK-01	0.041368	0.020165	0.061865	0.011549	0.059179	0.003551	0.069707	0.561361	0.012156	0.157901	0.269357	0.049038	0.048627	0.000159	0.000155	0.255036	0.045797	0	0.328104	0	0.019003	0.00034	0.069989	0.009667	50.11184
TCGA-G3-AAV7-01	0.059835	1.23093	0.11831	0.008299	0.357565	0.028365	0.242488	0.916323	0.042525	0.306138	0.709788	0.119283	0.08132	0.012121	0	0.135882	0.009946	0	0.747726	0	0.044344	0	0.16043	0.011115	188.8368
TCGA-ZP-A9CZ-01	0.08374	2.112289	0.073049	0.265634	0.234448	0.003879	0.075689	1.253114	0.007951	0.177007	0.288399	0.053198	0.033376	0.002138	0.000179	0.074586	0.024054	0	0.586211	0.000302	0.019038	0.00036	0.073547	0.002786	64.3281
TCGA-DD-A3A8-01	0.071239	0.126528	0.039443	0.016644	0.099376	0	0.058612	0.405913	0.000829	0.137566	0.427055	0.01565	0.014093	0.000491	0	0.135757	0.007858	0	0.317554	5.86E-06	0.004301	0	0.050763	0.004976	62.34539
TCGA-ED-A4XI-01	0.081625	0.481336	0.047471	0.119511	0.951539	0.029101	0.138025	1.990093	0.001665	0.231095	0.269322	0.575299	0.112099	0.009406	0.000147	0.255122	0.02802	0	0.742918	1.08E-05	0.015448	9.67E-06	0.076445	0.004589	68.91503
TCGA-DD-A1EG-01	0.068301	0.38937	0.117508	0.019477	0.327329	0.000981	0.037755	2.039078	0.00551	0.181602	0.465153	0.166326	0.039869	0.002383	0.000232	0.110966	0.001907	0	0.502085	4.26E-06	0.029632	0.000501	0.065986	0.005434	164.2606
TCGA-DD-A1EA-01	0.117589	0.332567	0.102709	0.046348	0.411864	0.000617	0.312611	2.028015	0.0002	0.302855	0.256008	0.041348	0.1662	0.000524	0.00017	0.067026	0.010071	0	0.708951	0.000106	0.010469	0.003745	0.186292	0.007972	63.05972
TCGA-DD-A1EI-01	0.051625	0.387433	0.134234	0.041647	0.290144	0.003815	0.03744	1.707752	0.009367	0.193561	0.300449	0.104529	0.034445	0.008219	0.000211	0.071105	0.007963	0	0.78212	0	0.018808	0	0.077105	0.004933	72.22808
TCGA-DD-A73E-01	0.070402	0.04983	0.107646	0.029618	0.097	0.000226	0.072603	0.459163	0.000172	0.263793	0.768691	0.019957	0.010326	0.001023	0	0.015014	0.026407	0	0.852962	6.87E-06	0.020365	0	0.164763	0.011667	47.35566
TCGA-DD-A113-01	0.059881	0.231816	0.135321	0.795805	0.186558	0.019321	0.011224	1.961541	0.005794	0.26284	0.232405	0.008538	0.00447	0.001555	0.000207	0.248076	0.160178	0	0.609265	0.000205	0.018086	0	0.099571	0.022565	39.89888
TCGA-DD-A115-01	0.05146	0.539703	0.057191	0.029182	0.182618	0.001382	0.105194	0.701527	0.010421	0.177235	0.243159	0.011835	0.106869	0.003526	0	0.070539	0.001882	0	0.954919	0.000393	0.014163	0.001482	0.048272	0.005958	64.0791
HCV 25 genes FPKM																									
Tags	PIGB	ITM2A	ANKRD27	RAB3D	OAS2	RASAL1	AIM1	STAT1	EGLN3	ZC3H4	AGAP3	IFI44L	HERC5	PRICKLE1	RSPH10B	B3GNT7	DEGS2	RSPH10B2	PARP14	GALNTL6	CCDC149	CSMD1	BCOR	RGS9BP	HLA-A
TCGA-FV-A4ZQ-01	0.113876	1.338657	0.118292	0.16207	0.421598	0.000345	0.166624	6.185951	0.000827	0.19503	0.355565	0.082245	0.026332	0.001125	0.000143	0.072362	0.002819	0	1.229186	0	0.004785	3.14E-06	0.077126	0.004463	268.8583
TCGA-WX-AA47-01	0.025159	0.021275	0.009312	0.003338	0.027089	0.0009	0.03668	0.142128	0.003203	0.082376	0.164402	0.002569	0.007693	5.10E-05	0	0.001198	0.001471	0	0.272069	0.003053	0.014862	0	0.022139	0	27.44041
TCGA-RG-A7D4-01	0.053985	0.625526	0.071706	0.031024	0.179928	0.001046	0.187445	2.293175	0.00048	0.147679	0.283997	0.063588	0.035595	0.000712	0.000694	0.086336	0.003417	0	0.882123	0	0.011602	0.001144	0.069622	0.008115	182.6273
TCGA-K7-A6G5-01	0.059009	0.264502	0.031527	0.019338	1.535745	0.002477	0.029603	2.263443	0.000492	0.081213	0.403755	0.750588	0.244597	0.001454	0.000587	0.069481	0.012042	0	0.692617	0	0.002168	6.43E-06	0.028816	0	185.4032
TCGA-2Y-A9HB-01	0.129969	0.513395	0.05204	0.025864	2.128863	0.001057	0.187741	3.816367	0.000747	0.213299	0.356518	2.135997	0.340748	0.001898	0.000146	0.064522	0.017274	0	1.716372	3.22E-05	0.007539	0.000147	0.042981	0.002279	172.0369
TCGA-K7-A5RG-01	0.050358	1.721901	0.095354	0.087415	0.633878	0.009305	0.285594	6.271143	0.008445	0.174478	0.33005	0.114353	0.072297	0.004037	0.000472	0.111231	0.004136	0.000157	0.945871	0	0.008817	0.002319	0.101648	0.009822	125.2293
TCGA-2Y-A9HA-01	0.052227	0.106135	0.091582	0.043766	0.446185	0.001476	0.190643	1.054013	0.00364	0.192736	0.427303	0.190147	0.093222	0.000711	0.000245	0.054022	0.001607	0	1.129524	0	0.03302	0.000311	0.101637	0.017173	43.65587
TCGA-MI-A75H-01	0.049708	0.297265	0.037761	0.016072	0.249825	0.000183	0.062131	0.502729	0.000266	0.130365	0.398251	0.118774	0.037044	0.002018	0	0.00972	0.002485	0	0.566864	5.56E-06	0.002354	0	0.020677	0.007081	105.9389
TCGA-ZP-A9D2-01	0.135762	9.000261	0.178043	0.048094	0.948933	0.077485	0.074839	10.41969	0.000997	0.350996	0.450531	0.603765	0.123575	0.016014	0.00058	0.161828	0.013333	0	1.230399	6.39E-05	0.032672	6.36E-06	0.132585	0.022617	78.03433
TCGA-KR-A7K2-01	0.02845	0.280825	0.058593	0.016332	1.257026	0.015297	0.104438	1.100206	0.001516	0.209231	0.391402	0.344429	0.147085	0.001849	0	0.049411	0.071042	0.000744	0.921009	0	0.017021	0.000237	0.02997	0.002909	135.1563
TCGA-K7-A5RF-01	0.061405	0.213895	0.040805	0.031704	3.973217	0.000814	0.181876	4.124384	0.001902	0.127355	0.331619	2.465718	0.507802	0.006483	0.000324	0.526932	0.048959	0	0.8038	1.19E-05	0.013277	0.000128	0.034418	0.007583	105.1071
TCGA-WX-AA46-01	0.041503	0.256662	0.031319	0.013645	0.138851	0.000145	0.137607	0.411376	0.000201	0.122447	0.238186	0.083896	0.057894	0.005706	0	0.04723	0.005125	0	0.566676	8.82E-06	0.011299	0	0.038119	0	22.70323
TCGA-2Y-A9H1-01	0.010713	0.104479	0.026689	0.010357	0.135497	0.001341	0.051664	0.395962	0.000916	0.059559	0.328061	0.03189	0.031885	0.000443	0	0.004463	0.004867	0	0.343023	0	0.012202	0.000219	0.015412	0.017338	103.4417
TCGA-MI-A75G-01	0.078257	0.114606	0.062534	0.00992	0.059581	0	0.079002	0.447695	0.000376	0.156171	0.658357	0.006994	0.083425	0.000144	0	0.016873	0.027606	0.000419	0.402067	5.14E-06	0.00178	0.001194	0.032931	0.004371	33.01003
TCGA-MI-A75E-01	0.053905	0.25411	0.041095	0.021195	0.246866	0.006098	0.082322	0.741468	0.000953	0.119574	0.491803	0.042784	0.075217	0.004273	0.00014	0.074415	0.029976	0	0.642805	1.03E-05	0.005826	6.16E-06	0.027291	0	76.21657
TCGA-WX-AA44-01	0.062669	0.174686	0.098165	0.011979	0.401531	0.064128	0.030858	0.612469	0.003627	0.359845	0.459745	0.204298	0.095351	0.003889	0.000989	0.651186	0.012181	0	1.018519	0	0.050394	5.42E-06	0.094708	0.01157	180.3069
TCGA-KR-A7K0-01	0.052633	0.118602	0.057555	0.016182	0.346983	0.002328	0.094033	0.780946	0.004165	0.16609	0.410016	0.13454	0.062906	0.002035	0.000643	0.042613	0.013204	0	0.687904	0	0.007298	0.000384	0.051352	0.005017	87.58488
TCGA-GJ-A3OU-01	0.045661	1.053112	0.093055	0.069154	0.54144	0.012566	0.281713	2.380869	0.014244	0.18678	0.540439	0.110206	0.048138	0.007569	0	0.40602	0.018532	0	0.895155	1.59E-05	0.019584	4.76E-06	0.058005	0	202.934
