# Supplementary material for: A comprehensive genome-wide profiling comparison between HBV and HCV infected hepatocellular carcinoma
Source: BMC Med Genomics. 2019 Oct 28;12:147. doi: 10.1186/s12920-019-0580-x (PMC6819460; doi:10.1186/s12920-019-0580-x)
Supplement: Supplementary file 5 — Additional file 5: Figure S1. The Circos plot of differential methylation in HCV-infected compared with HBV-infected HCCs. The outer layer represents CpG islands heatmap. Red color represents CpG islands are hypermethylated. Blue color represents CpG islands are hypomethylated. The middle layer represents the scatter plot of a single CpG site. Each dot represents a significant different DNA methylation changes, with p-value associated at Y-axis. The inner layer of circular plot is top 25 genes with the largest betafc value in DNA methylation changes of CpG islands. [file 12920_2019_580_MOESM5_ESM.pdf]

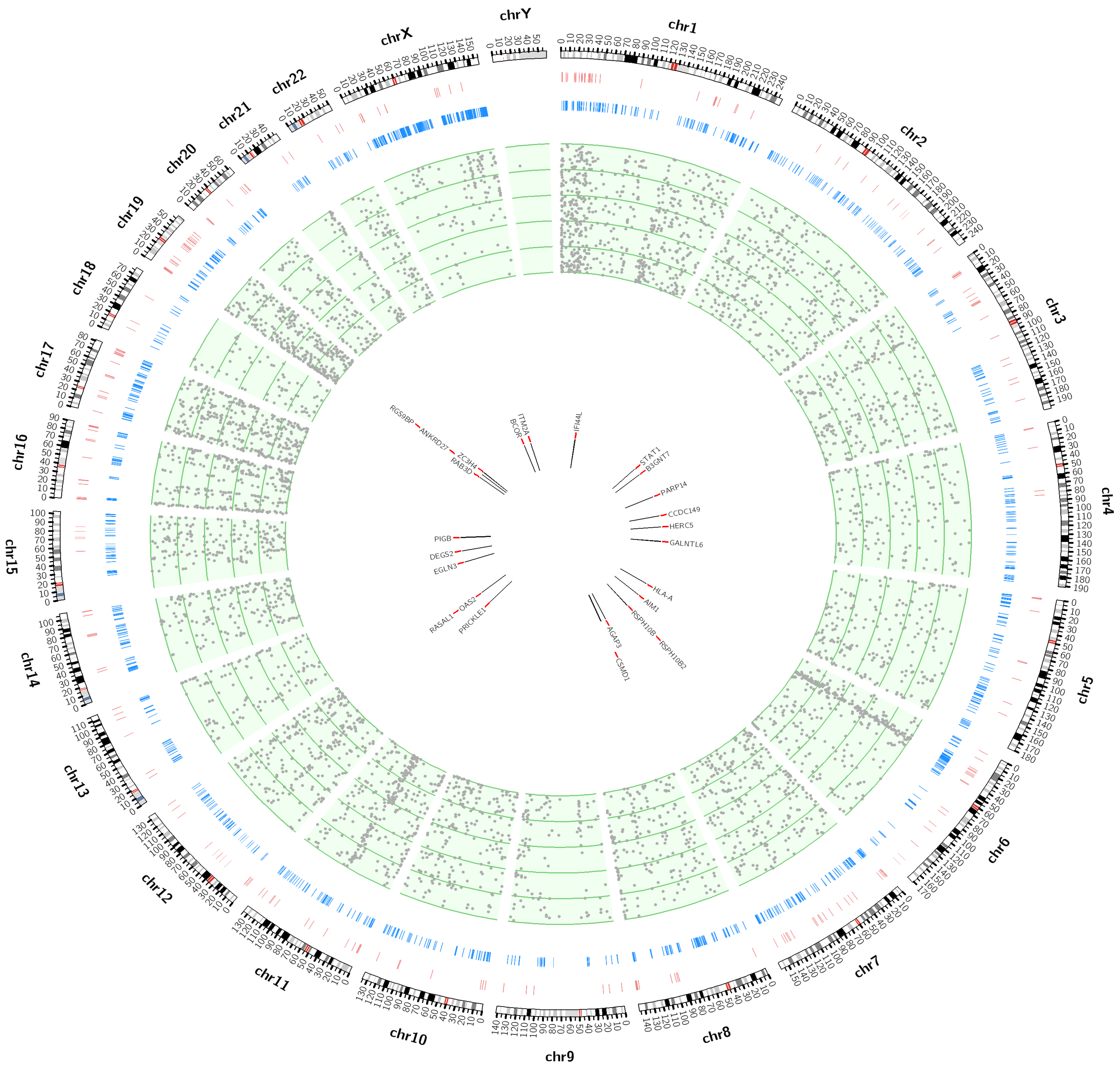

Figure S1. Circos Plot of differential methylation in HCV-infected compared with HBV-infected HCCs

The outer layer represents CpG islands heatmap. Red color represents CpG islands are hypermethylated. Blue color represents CpG islands are hypomethylated. The middle layer represents scatter plot of single CpG site. Each dot represents a significant different DNA methylation changes, with p-value associated at Y-axis. The inner layer of circular plot is top 25 genes with the largest betafc value in DNA methylation changes of CpG islands.
