# Supplementary figures and images for: A comprehensive genome-wide profiling comparison between HBV and HCV infected hepatocellular carcinoma
Source: BMC Med Genomics. 2019 Oct 28;12:147. doi: 10.1186/s12920-019-0580-x (PMC6819460; doi:10.1186/s12920-019-0580-x)

(A)

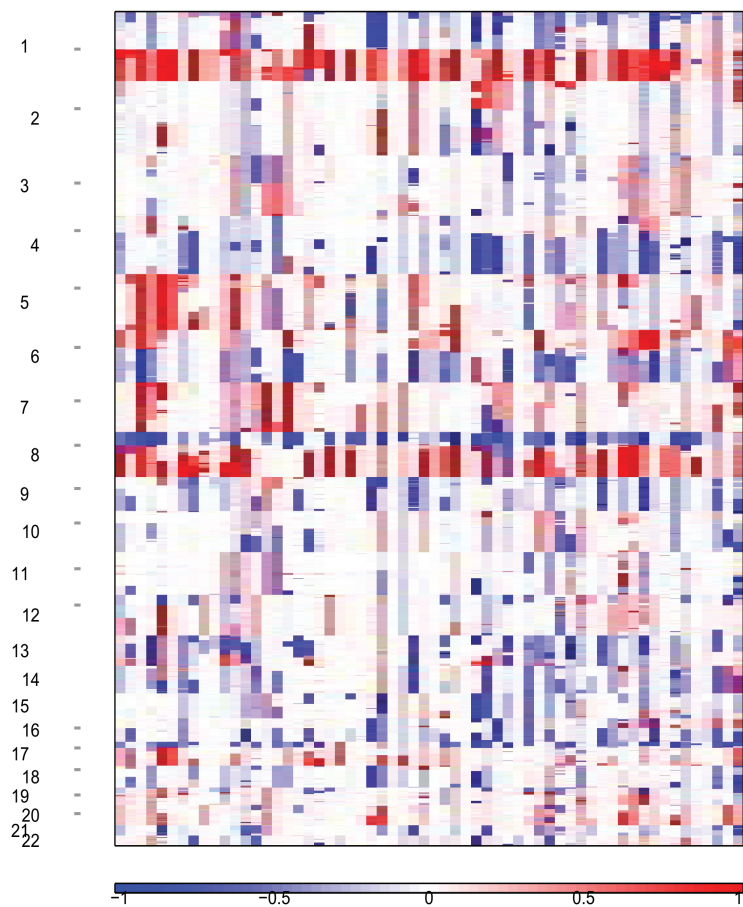

(B)

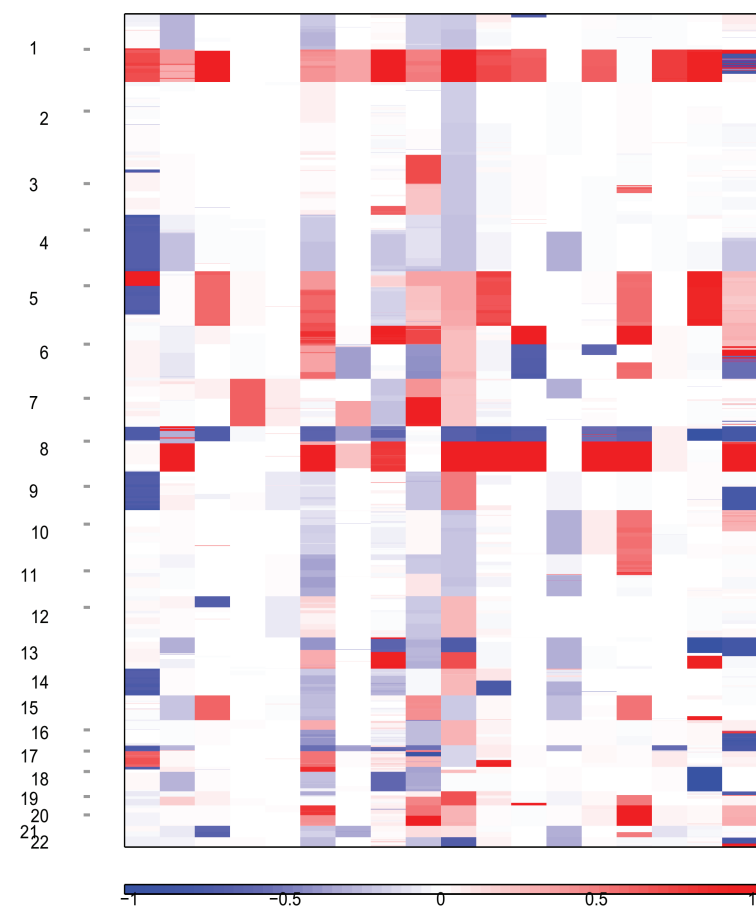

Figure S2. Heatmap of copy number variation in HBV (A) and HCV (B)

Supplement: Supplementary file 6 — Additional file 6: Figure S2. Heatmap of copy number variation in HBV (A) and HCV (B). [file 12920_2019_580_MOESM6_ESM.pdf]
